# Fate of biosolids‐bound PFAS through pyrolysis coupled with thermal oxidation for air emissions control

**DOI:** 10.1002/wer.11149

**Published:** 2024-11-12

**Authors:** Lloyd J. Winchell, Joshua Cullen, John J. Ross, Alex Seidel, Mary Lou Romero, Farokh Kakar, Embrey Bronstad, Martha J. M. Wells, Naomi B. Klinghoffer, Franco Berruti, Alexandre Miot, Katherine Y. Bell

**Affiliations:** ^1^ Brown and Caldwell Walnut Creek California USA; ^2^ Department of Chemical and Biochemical Engineering, Institute for Chemicals and Fuels from Alternative Resources (ICFAR) Western University London Ontario Canada; ^3^ EnviroChem Services Cookeville Tennessee USA; ^4^ Silicon Valley Clean Water Redwood City California USA

**Keywords:** biosolids, PFAS, pyrolysis, thermal oxidizer, wastewater

## Abstract

Pyrolysis has been identified as a possible thermal treatment process for reducing perfluoroalkyl and polyfluoroalkyl substances (PFAS) from wastewater solids, though off‐gas from the pyrolysis unit can still be a source of PFAS emissions. In this work, the fate of PFAS through a laboratory‐scale pyrolysis unit coupled with a thermal oxidizer for treatment of off‐gasses is documented. Between 91.5% and >99.9% reduction was observed through the entire system for specific compounds based on targeted analyses. Overall, the pyrolysis and thermal oxidizer system removed 99.4% of the PFAS moles introduced. Furthermore, shorter chain variants comprised the majority of reportable PFAS in the thermal oxidizer flue gas, indicating the longer chain compounds present in the dried biosolids fed to pyrolyzer decompose through the system.

**Practitioner Points:**

- Thermal oxidation is a promising treatment technology for exhaust systems associated with thermal biosolids treatments.
- Thermal oxidation demonstrated significant degradation capabilities, with gas phase emissions comprising only 0.200% of initial PFAS concentrations to the system.
- Short‐chain PFAS made up a higher percent of thermal oxidizer emissions, ranging between 54.4% and 79.5% of PFAS in the exhaust on a molar basis.
- The possibility of recombinant PFAS formation and partial thermal decomposition of PFAS in thermal oxidation is a needed area of research.

## Graphical abstract

A laboratory‐scale pyrolysis and thermal oxidizer system emitted 0.200% of PFAS moles present in wastewater biosolids processed by the system to the atmosphere. Biochar contained 0.442% of the feed PFAS on a similar basis. Pyrolysis with thermal oxidation is a promising PFAS treatment technology for wastewater applications.

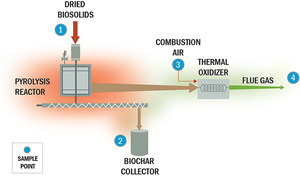

## INTRODUCTION

Although they have been in use for several decades, perfluoroalkyl and polyfluoroalkyl substances (PFAS) have been recently identified as a significant environmental contaminant of concern (Fenton et al., 2021). Their pervasive use in a myriad of industrial processes and domestic products means that PFAS are continually discharged to and present in both the liquids and solids streams of wastewater treatment plants (Behnami et al., 2024; Kurwadkar et al., 2022; Oza et al., 2024; Schaefer et al., 2023). The presence of PFAS in wastewater sludges (unstabilized waste solids residuals from the treatment process) and biosolids (stabilized solids) has instigated an international research focus on detection, quantification, and treatment of these constituents, focusing primarily on thermal treatment (Hušek et al., 2024; Longendyke et al., 2022; Winchell et al., 2022a, 2024; Winchell, Ross, et al., 2021; Winchell, Wells, et al., 2021). Thermal degradation of PFAS in wastewater solids (inclusive of sludges and biosolids) has been demonstrated, and the extent of that degradation in the various thermal processes requires further investigation (Winchell et al., 2024).

Pyrolysis is a proven technology for processing lignocellulosic biomass at high temperatures in the absence of oxygen to generate a fuel‐rich off‐gas, bio‐oil, and biochar (Cha et al., 2016). Pyrolysis is also emerging as a treatment alternative for wastewater solids for potential beneficial reuse of the residual biochar (Ihsanullah et al., 2022; McNamara et al., 2023; Winchell et al., 2022b; Xing et al., 2021), as well as a possible reduction in PFAS content. Research regarding the removal efficiency of PFAS from biosolids through pyrolysis has focused largely on the solid fraction of the pyrolysis products, the biochar itself. Pyrolysis for biosolids treatment has been suggested to alleviate the human and environmental PFAS exposure risk resulting from land application of biosolids (Bamdad et al., 2022; Johnson, 2022; Pepper et al., 2021). However, questions still remain regarding the fate and transformation of PFAS in the exhaust gasses of pyrolysis treatment of wastewater solids.

Studies that have sought to ascertain the fate of PFAS through pyrolysis of biosolids still recognize knowledge gaps regarding the fraction and type of PFAS volatilized (and emitted) in the pyrolysis off‐gas (Longendyke et al., 2022; McNamara et al., 2022, 2023; Thoma et al., 2022). The documented presence of PFAS in scrubber water treating thermal oxidizer flue gas that combusts the pyrolyzer off‐gas (Thoma et al., 2022) indicates that PFAS or their partial decomposition products volatilize and exit the thermal oxidizer. Sørmo et al. (2023) detected PFAS in flue gas of combusted pyrolysis off‐gas, referred to as “syngas.” The study further hypothesized that the presence of PFAS in flue gas of combusted pyrolysis off‐gas was representative of full‐scale pyrolysis units in general but could not definitively draw conclusions due to the lack of studies evaluating this process (Sørmo et al., 2023). Sørmo et al. (2023) further suggested that off‐gas combustion design should be a focus when optimizing PFAS destruction in pyrolysis applications. Hušek et al. (2024) also detected PFAS in condensing vapors from a pyrolysis reactor treating wastewater sludges, noting a decline in the amount of PFAS collected in the off‐gas condenser at higher pyrolysis temperatures (500°C). The authors attributed the decline to a greater degree of mineralization or cleavage into PFAS that was not monitored as part of the campaign.

One potential option for addressing PFAS in pyrolysis off‐gas centers on emissions treatment. Demonstration and commercial scale pyrolysis systems often use thermal oxidizers to combust the pyrolysis off‐gas from which the resulting heat can be used to support the process (Winchell et al., 2022a). Thermal oxidizers typically operate at temperatures of 850°C or higher for more than 2 s and can achieve a greater degree of combustion efficiency than furnace technologies typically employed to process multi‐phase material such as wastewater solids within a single reactor (Winchell, Ross, et al., 2021) In contrast, pyrolysis reactors are designed to exclude oxygen and, by processing the gas through a thermal oxidizer, more efficiently blend the combustible off‐gas with oxygen within a short retention time, requiring less excess air than necessary for combustion. As shown by McNamara et al. (2022), transfer of PFAS from the biosolids feedstock in a pyrolysis reactor to the off‐gas warrants use of a thermal oxidizer for PFAS treatment. For these reasons, thermal oxidizers have been permitted in the manufacturing industry as the best available control technology (BACT) for PFAS treatment from emissions at the Saint‐Gobain Performance Plastics (SGPP) and Chemours facilities (Beahm, 2019; Focus Environmental Inc, 2020). By permit, the facilities must meet minimum combustion temperatures of 1,000°C (State of New Hampshire Department of Environmental Services Air Resources Division, 2023) and 980°C (North Carolina Department of Environmental Quality, 2020), for the SGPP and Chemours facilities, respectively. At 1,000°C, the regenerative thermal oxidizer at SGPP achieved 92% and 75% removal for PFOA and PFOS, respectively (BARR Engineering Company, 2022). The Chemours thermal oxidizer has been operated hotter than required by permit and recently achieved greater than 99.999% removal of five fluorinated compounds while operating at 1,100°C (Focus Environmental Inc., 2022). The critical step for achieving PFAS control in pyrolysis systems, therefore, is likely the design and operation of the downstream thermal oxidizer.

There is growing evidence for thermal oxidizers as a strategy for PFAS destruction in pyrolysis off‐gas (Sørmo et al., 2023; Winchell et al., 2022b). On the contrary, relatively little information exists on both PFAS levels in pyrolysis off‐gas and holistic mass balancing of PFAS through pyrolysis units coupled to thermal oxidizers. This study contributes to the burgeoning library of research and empirical knowledge on this particular aspect of pyrolysis of wastewater solids. For this evaluation, dried biosolids were processed through a laboratory pyrolysis reactor coupled to a thermal oxidizer with operating conditions resembling an existing full‐scale process. This approach was used to validate laboratory‐scale PFAS destruction performance and could provide replicate data for full‐scale operations. Samples of dried biosolids and biochar, in addition to combustion air and flue gas from the thermal oxidizer, were collected for targeted PFAS analysis to characterize PFAS fate through the system. Data were used to demonstrate whether targeted PFAS sequester in the biochar or volatilize in the off‐gas, identify transformation products of PFAS that may be produced as a consequence of thermal treatment, and further elucidate the application of thermal oxidation as a technology for preventing PFAS from being emitted from pyrolysis units.

## METHODOLOGY

This project characterized the fate of targeted PFAS in a laboratory reactor system that aims to replicate the large‐scale municipal biosolids pyrolysis system at Silicon Valley Clean Water (SVCW) in Redwood City, CA, United States of America (USA). The laboratory reactor temperatures and retention times were selected to mimic the SVCW full‐scale operating conditions for pyrolysis and thermal oxidation.

### System description

In 2021, Western University Institute for Chemicals and Fuels from Alternative Resources (ICFAR) fabricated a solids pyrolysis and thermal oxidizer laboratory reactor system with the ability to pyrolyze a variety of feedstocks, including wastewater solids over a range of operating conditions and process the resulting off‐gas stream with a condenser assembly to produce oil or with a thermal oxidizer for emissions control and heat production. The system consists of six sections including a feeding system, a pyrolysis reactor, a thermal oxidizer, a biochar extractor, a condensing unit, and a gas extractor exhaust line, depicted in Figures S1 and S2, respectively.

Two kilograms of dried biosolids feedstock were added to the feed hopper that metered material into the pyrolysis reactor for each experimental run. Dried biosolids were continuously fed to the pyrolysis reactor from the feed hopper, and biochar was intermittently extracted using screw augers. The feed hopper head space was purged with nitrogen gas. The pyrolysis solids residence time was calculated by dividing the reactor bed volume by the combined feed and discharge rate, accounting for mass reduction within the reactor. Pyrolysis off‐gasses were directed to a thermal oxidizer installed inside an electrical furnace. The thermal oxidizer gas residence time represents the reactor volume divided by the measured flue gas flow rate.

Prior to introducing dried biosolids, the pyrolysis reactor was brought to the desired operating temperature, which was maintained through an electrical conduction band heater. The internal thermal oxidizer temperature was brought to approximately 950°C using the electrical heating system, and the combustion of the pyrolysis off‐gas raised this to the reported run temperature. Flue gas flow rate was continuously measured using an Omega FMA series bottle air flow meter (Omega Engineering, Inc., Norwalk, Connecticut, USA), and oxygen content was calculated based on air input and complete combustion of volatile compounds and gasses introduced to the thermal oxidizer.

Table 1 provides an overview of the operating conditions for the laboratory‐scale system. Additionally, Table 2 summarizes the operational characteristics of three experimental runs including feed rate of dried biosolids, biochar yield, flue gas flow rate, and air supply to the thermal oxidizer.

**TABLE 1:** Laboratory‐scale system operating conditions

Process	Parameter	Value	Comments
Pyrolysis	Solid retention time	75 min	
	Gas residence time	Negligible	Gas was transferred to thermal oxidizer with minimal transfer line length
	Operating temperature	600°C	
Thermal oxidizer	Temperature	1,000°C	
	Residence time	2 s	
	Excess oxygen (λ_C_ )	1.5–2.0	Estimated

**TABLE 2:** Experimental run summary

Parameter	Experimental Run 1	Experimental Run 2	Experimental Run 3
Dried biosolids feed rate to pyrolysis, kg/h	0.260	0.283	0.334
Dried biosolids total feed, kg	1.31	1.49	0.969
Run time, h	5.0	5.0	2.90
Biochar collected, kg	0.518	0.567	0.311
Pyrolysis vapor and gasses feed rate to the thermal oxidizer, kg/h of volatiles	0.156	0.169	0.210
Air fed into the thermal oxidizer, L/min	15	15	15
λ_C_	2.0	1.85	1.48
Average flue gas flowrate, L/min	18.3	18.8	20.2

*Note*: λ_C_ = stoichiometric ratio of oxygen supplied versus the combustion reaction requirements.

Thermal oxidizer turbulence is not characterized in Tables 1 and 2 and was not part of this research. Although assessment of gas‐phase turbulence can be performed using computational fluid dynamics, this investigation did not include such an analysis. Turbulence is one of the three critical parameters for combustion efficiency, along with temperature and residence time. Future research should evaluate turbulence based on results presented here.

### Sampling, sample preparation, and analysis

A comprehensive analytical workflow aimed at achieving a mass balance of targeted PFAS across gaseous and solid matrices encountered in the pyrolysis and thermal oxidizer units was designed. While a strict mass balance is not possible with current analytical techniques (Winchell, Wells, et al., 2021), a mass balance approach was used to assess targeted, quantifiable PFAS listed in Section S3.3.

All major inputs and outputs (e.g., dried biosolids, combustion air, biochar, and flue gas) were sampled in triplicate. Dried biosolid samples from SVCW were collected and shipped to ICFAR in London, Ontario (Canada), where the samples were processed through the laboratory‐scale continuous pyrolysis system. Sampling was conducted on May 30, June 1, and June 2 of 2023. Additional details of the sampling events and samples collected are provided in Section S3.5. Samples collected during the events are summarized in Table S1.

**FIGURE 1:**
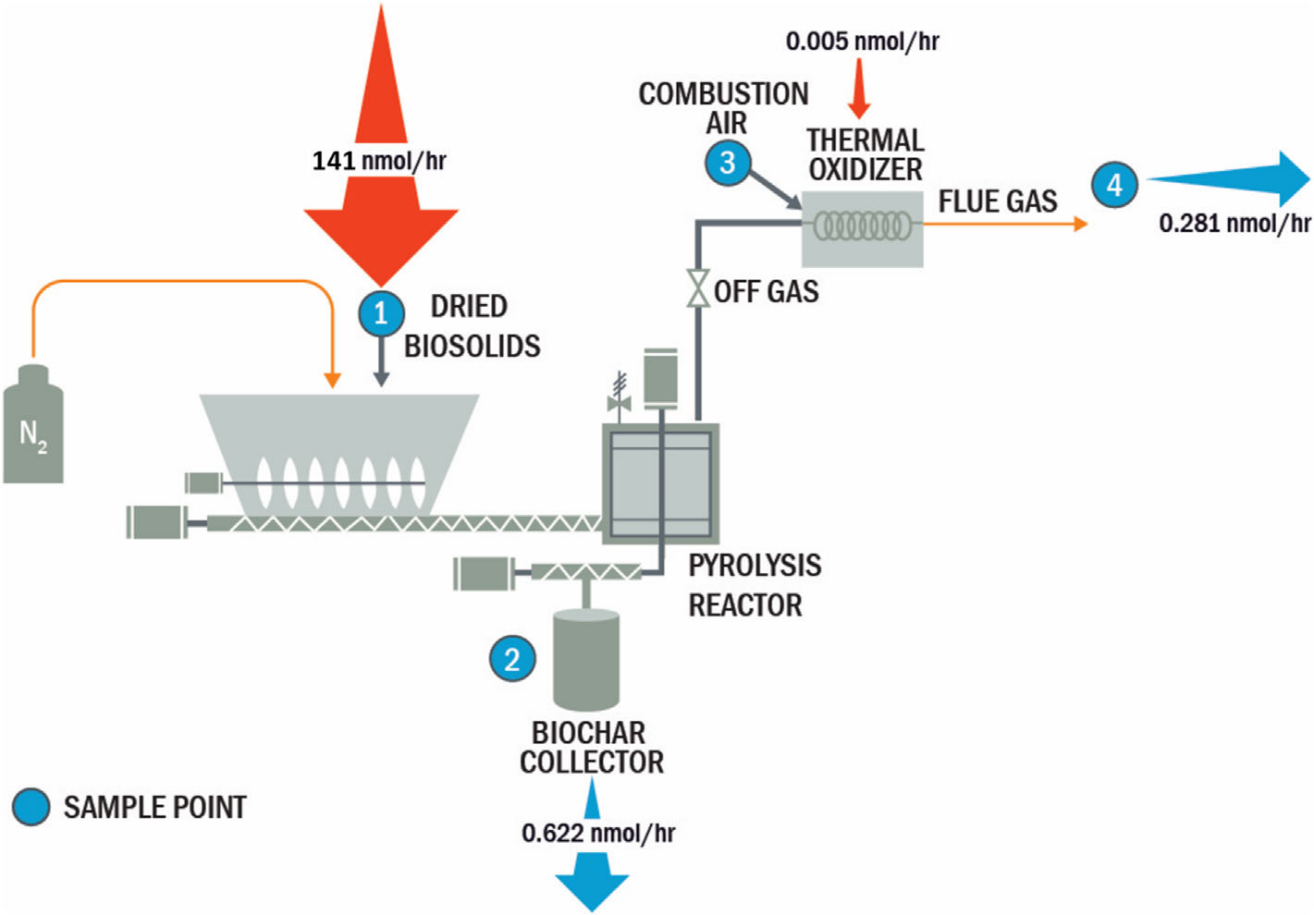
Averaged results of triplicate runs through the pyrolysis system

Figure 1 graphically depicts the laboratory‐scale system and sampling points. Additional details are provided in the Sections S1 and S2.

On‐site sampling was supervised by personnel from ICFAR and ORTECH Consulting Inc (ORTECH), Mississauga, Ontario, Canada. Flue gas samples were collected by ORTECH, while dried biosolids and biochar samples were collected by ICFAR. Samples were analyzed by Eurofins Test America (ETA), Knoxville, TN, USA, and Lancaster, PA, USA.

### Flue gas emissions

The United States Environmental Protection Agency's (USEPA, 2021) “Other Test Method 45 (OTM‐45) Measurement of Selected Per‐ and Polyfluorinated Alkyl Substances from Stationary Sources” was referenced to sample flue gas emissions from the thermal oxidizer (Figures S3 and S4). Refer to Section S3.1 for additional details on the four fractions—front half, back half, impinger condensate, and breakthrough XAD resin—collected from the sampling train. A field blank train (FBT) and proof blank train (PBT) were collected for quality control. The FBT and PBT use the same assembly as the sample train. The trains are assembled at the site, heated, and leak checked, but no sample is pulled through. The PBT uses clean glassware from the laboratory, while the FBT uses glassware previously used to collect a sample at the site and has been rinsed. Approximately 3 L/min of the thermal oxidizer flue gas was processed through the sampling train for 2.5–5.0 h, depending on the run. Targeted analytical results from each fraction are summed to represent the flue gas PFAS levels.

Samples collected with the OTM‐45 sample train were extracted using a sequential procedure. Surrogate and isotope dilution standards were spiked into samples prior to laboratory extraction. Field rinses of the sample train components were performed with ammonium hydroxide (NH_4_OH) in methanol (CH_3_OH) and were collected in separate sample containers. Each sample train rinse was combined with the extract of the parent fraction. For the liquid impinger samples, the polar analytes were extracted with a mixed‐mode solid‐phase extraction/weak anion exchange SPE/WAX) sorbent. The other samples, with exception of impinger condensates, were extracted with a NH_4_OH in CH_3_OH solution.

### Combustion air

Combustion air supplied to the thermal oxidizer originated from the laboratory atmosphere. The combustion air sampling methodology is described in Section S3.2. An XAD sorbent was used to collect the combustion intake air sample (Figure S5). Samples were collected using the same apparatus and sample preparation as used for the breakthrough XAD‐2 module in the flue gas sampling train (Figure S4). Approximately 3 L/min of combustion air was processed through the apparatus for 2.5–5.0 h concurrently with the flue gas sampling.

### Dried biosolids and biochar

A grab sample of homogenized dried biosolids was collected prior to being fed into the pyrolysis reactor, and a grab sample of the biochar produced was collected for analysis. Both the dried biosolids and biochar contained no filterable liquid phase. These samples were processed in the same manner and surrogate and isotope dilution standards were spiked into samples prior to extraction. An aliquot of both matrices was collected for total solids (TS) and volatile solids (VS) analyses. Raw samples were extracted with a 0.4% potassium hydroxide (KOH) and CH_3_OH solution subjected to shaking and sonication with final pH adjustment. The extract was cleaned using SPE/WAX with graphitized carbon black. PFAS were eluted using 0.3% NH_4_OH and CH_3_OH solution. The extracts were analyzed chromatographically by polar targeted analysis.

### Analytical methods

At the time this project was proposed and funded (spring 2023), the USEPA had promulgated two methods (537.1 and 533, Shoemaker & Tettenhorst, 2020) for quantifying PFAS, mainly polar compounds, in drinking water. However, draft method USEPA Draft Method 1633 (to test for 40 PFAS compounds in wastewater, surface water, groundwater, soil, biosolids, sediment, landfill leachate, and fish tissue) was referenced prior to being finalized in January 2024 (USEPA, 2024a). The following identifies the standard methods, modifications thereof, or emerging techniques utilized in this research.

#### Targeted PFAS

Quantitative targeted analyses utilized known reference standards, based on standard regulatory methods (Shoemaker & Tettenhorst, 2020) and extended methods established by the analytical vendor referred to here as Method 537 (modified). The list of PFAS examined is presented in Table S2. Refer to this table for the names and acronyms of individual PFAS compounds and the PFAS families to which they belong, hereafter referred to by their acronyms (Section S3.3). The commercial information for the reagents and instruments can be found in Table S3.

#### Solid characteristics

The dried biosolids and biochar were characterized as described in Section S3.4. Each sample was analyzed for moisture and volatile content using Standard Methods 2540G.

#### Data quality standards

Project analytical data were assessed for data quality in a four‐step process outlined in Winchell et al. (2024). In short, after confirming the data is that collected for the study, data containing a laboratory qualifier (Table S4) are screened from evaluation, so any findings or conclusions are stated with high confidence.

## RESULTS

The data presented here have passed the quality review process and deemed usable for interpretation. Forty‐nine specific PFAS were analyzed, although not all PFAS were evaluated for each sample (refer to Table S2 for analyte lists). PFAS were evaluated as individual compounds or grouped into long‐ or short‐chain categories, following guidelines of others (Buck et al., 2011), to assess fate and potential degradation trends based on composition. See Section S4 for more information defining long‐ and short‐chain qualities.

Potential inputs of PFAS entering the pyrolysis system include the dried biosolids feed to the pyrolyzer and combustion air supplied to the thermal oxidizer. Potential outputs of PFAS from the pyrolysis system include the biochar leaving the pyrolyzer and flue gas exiting the thermal oxidizer. Across all samples, the family of PFAS with the largest reportable representation was the perfluoroalkyl carboxylic acids as discussed in the following. The reportable results from the analysis of these samples are presented in Tables 3 and 4; all data are reported in Tables S5–S13.

**TABLE 3:** Pyrolysis system PFAS inputs

Family	PFAS acronym	Dried biosolids (Run 1/2a/2b/3)^a^		Combustion air (Run 1/2/3)	
		Concentration	Load	Concentration	Load
	–	ng/g dry	ng/run	ng/sample	ng/run
PFCA	PFBA	Q/Q/Q/Q	Q/Q/Q/Q	Q/Q/Q	Q/Q/Q
	PFPeA	2.33/2.78/3.16/Q	3,060/4,140/4,710/Q	Q/Q/Q	Q/Q/Q
	PFHxA	16.4/19.5/17.6/16.9	21,500/29,100/26,300/16,400	Q/Q/Q	Q/Q/Q
	PFHpA	4.93/6.23/4.93/5.06	6,470/9,290/7,350/4,900	Q/Q/Q	Q/Q/Q
	PFOA	92.4/99.3/96.2/92.8	121,000/148,000/143,000/89,900	3.88/1.28/1.29	15.5/ 4.99/4.87
	PFNA	3.38/3.82/3.36/3.22	4,440/5,700/5,010/3,120	Q/Q/Q	Q/Q/Q
	PFDA	11.1/13.8/11.9/12.2	14,500/20,600/17,700/11,800	Q/Q/Q	Q/Q/Q
	PFUnA	1.73/Q/Q/Q	2,270/Q/Q/Q	Q/Q/Q	Q/Q/Q
	PFDoA	Q/Q/3.86/4.02	Q/Q/5,760/3,890	Q/Q/Q	Q/Q/Q
PFSA	PFHpS	25.7/25.7/22.6/27.1	33,700/34,800/33,700/26,200	Q/Q/Q	Q/Q/Q
FOSE	NEtFOSE	Q/Q/Q/2.82	Q/Q/Q/2,730	Q/Q/Q	Q/Q/Q
	NMeFOSA	14.2/Q/Q/Q	18,600/Q/Q/Q	Q/Q/Q	Q/Q/Q

Abbreviation: Q, data screened during quality control.

^a^ Run 2b is a field duplicate of Run 2a and used for data quality control only.

**TABLE 4:** Pyrolysis system PFAS outputs

Family	PFAS	Run 1/2/3						
		Biochar		Exhaust				
		Concentration	Emission	Front half	Back half	Impingers	Breakthrough	Emission
	–	ng/g dry	ng/run	ng/sample	ng/sample	ng/sample	ng/sample	ng/run
Fluorotelomers	6:2 FTS	Q/Q/Q	Q/Q/Q	5.05/Q/44.2	Q/Q/Q	Q/Q/Q	Q/Q/Q	31.4/Q/291
PFCA	PFBA	Q/Q/Q	Q/Q/Q	Q/Q/Q	Q/20.7/Q	Q/Q/Q	Q/Q/Q	Q/128/Q
	PFPeA	0.716/0.387/0.170	371/217/53.3	1.95/1.45/3.1	3.28/1.25/2.93	1.23/0.634/Q	Q/Q/Q	40.1/20.7/39.7
	PFHxA	Q/0.474/Q	Q/266/Q	3.72/3.42/6.06	7.42/Q/3.72^a^	Q/Q/Q	Q/Q/Q	69.2/21.2/64.4
	PFHpA	0.205/0.138/Q	106/77.5/Q	2.43/1.41/3.61	4.96/3.73/3.67	Q/Q/Q	Q/Q/Q	45.9/31.9/47.9
	PFOA	0.953/0.548/0.234	494/308/73.4	9.12/7.61/10.5	Q/Q/Q^b^	Q/Q/Q	Q/2.56/1.18	56.7/63.1/76.9
	PFNA	Q/Q/Q	Q/Q/Q	1.98/1.17/1.41	1.13/Q/1.02	Q/Q/Q	Q/Q/Q	19.3/72.5/16.0
	PFDA	Q/0.122/Q	Q/68.5/Q	1.28/Q/Q	Q/Q/Q	Q/Q/Q	Q/Q/Q	7.95/Q/Q
	PFUnA	Q/Q/Q	Q/Q/Q	Q/Q/Q	Q/Q/1.08	Q/Q/Q	Q/Q/Q	Q/Q/7.11
PFSA	PFHpS	2.05/Q/Q	1,060/Q/Q	Q/Q/Q	Q/Q/Q	Q/Q/Q	Q/Q/Q	Q/Q/Q
FOSE	NEtFOSE	0.206/Q/Q	107/Q/Q	Q/Q/Q	Q/Q/Q	Q/Q/Q	Q/Q/Q	Q/Q/Q
	NMeFOSA	0.522/Q/Q	Q/Q/Q	13.1/9.60/5.63	Q/Q/Q	Q/Q/Q	Q/Q/Q	81.4/59.5/37.1
FOSAA	NMeFOSAA	0.215/Q/Q	270/Q/Q	Q/Q/Q	Q/Q/Q	Q/Q/Q	Q/Q/Q	Q/Q/Q

Abbreviation: Q, data screened during quality control.

^a^ The FBT sample analysis produced a J qualified value; reportable run data were not screened.

^b^ Reportable run data screened due to the contamination in the FBT.

### Inputs

#### Dried biosolids

On a molar basis, perfluoroalkyl carboxylic acids accounted for an average of 70.8% of PFAS observed across all three runs, or 432 of the 609 nmol fed to the system, with the largest contribution coming from PFOA (47.5% of observed on a molar basis). The next largest contributors were PFHxA, a short‐chain perfluoroalkyl carboxylic acid, and PFHpS, a long‐chain perfluoroalkyl sulfonic acid, each of which contributed 11.6% of the observed PFAS on a molar basis. The other PFAS observed in this sample included NEtFOSAA and NMeFOSAA, in all three runs, and NMeFOSA and NEtFOSE, only in the first and third runs, respectively. The particularly high concentration of PFOA contributed to the preponderance of long‐chain PFAS, which represented between 85.5% and 88.4% of the PFAS observed in the dried biosolids.

#### Ambient air

Ambient air was collected for analysis to characterize the combustion air used in the thermal oxidizer coupled to the pyrolyzer. The only PFAS with reportable results in this sample was PFOA, which was detected in all three runs. This represented only 0.00352%, or 0.0204 nmol introduced to the thermal oxidizer on average, of the total PFAS measured entering the system on a molar basis.

### Outputs

#### Biochar

Of all PFAS studied, only two perfluoroalkyl carboxylic acids were observed in reportable amounts for all three runs in this sample: one long‐chain (PFOA) and one short‐chain (PFPeA). The first run represented the largest concentration of biochar‐bound PFAS on a molar basis, as well as the largest number of PFAS with reportable results. Perfluoroalkyl carboxylic acids represented 47.3%, or 2.89 nmol in the biochar on average, on a molar basis of reportable PFAS. PFHpS, NEtFOSE, NMeFOSA, and NMeFOSAA were also observed in this first run with PFHpS exceeding all other PFAS reported. All the PFAS observed for the second and third runs came from the perfluoroalkyl carboxylic acid family.

#### Thermal oxidizer flue gas

The largest contributors to the observed amounts of PFAS in this sample were also from the perfluoroalkyl carboxylic acid family. On a molar basis, this family accounted for 73.8% and 90.0% of the reportable PFAS for Runs 1 and 2 of 0.824 and 1.04 nmol emitted during the run, respectively, but only 46.7% of 1.40 nmol for the third run. 6:2 FTS was reportable in Runs 1 and 3 only, and in Run 3 made up 48.6% on a molar basis of the reportable PFAS. The only other PFAS observed in reportable amounts was NMeFOSA, observed in all three runs. Of the perfluoroalkyl carboxylic acids observed, the majority (on a molar basis) were short‐chain PFAS (61.3%, 79.5%, and 54.4% for Runs 1–3, respectively). No PFAS was reportable in all four OTM‐45 fractions. The front half, particulate in nature, and back half fractions on average captured 46.4% and 34.1% of the molar PFAS collected in the sample trains; however, the range of capture among the runs was high: for the front half, the percent capture ranged from 34.5–67.5% across the runs, while for the back half, the percentages ranged from 12.7–60.4%.

## DISCUSSION

Performance of pollutant reductions through a process is generally characterized from a destruction and removal efficiency (DRE) perspective. However, DRE for PFAS must be carefully evaluated and described to avoid misleading characterizations. Full‐scale PFAS DRE remains elusive when applying appropriate data usability guidelines, previously referenced. Moreover, a complete mass balance based on organic fluorine was not attempted with the analysis performed for this study, as total organic fluorine (TOF) and volatile fluorinated compound (VFC) byproduct analyses were not conducted. An inorganic fluorine balance may be possible as well given the laboratory system is constructed of all metal equipment with no cementitious refractory lining that may react with hydrofluoric acid produced from PFAS thermal decomposition (Winchell, Ross, et al., 2021). However, data gathered were used to develop an assessment of targeted PFAS summed on a molar basis, depicted in Figure 1. Values presented in Figure 1 represent the average molar load of all targeted PFAS summed during each of three sampling runs presented on an hourly rate. This represents a nearest approach to a mass balance or DRE.

The majority of PFAS observed (>99.9%) entered the pyrolysis system in the dried biosolids. Of those analytes, during the three runs, removal efficiencies between 91.5% and 100% for individual compounds were observed, with the majority of the emitted PFAS (68.9% molar basis) observed being present in the biochar as opposed to the thermal oxidizer flue gas.

### Biosolids and biochar

Between the input of dried biosolids and the production of biochar, a reduction of 99.0–99.9% was observed for targeted PFAS entering the system. The largest contribution came from PFHpS accounting for 0.47 nmol/h, or 38.7%, of the biochar PFAS, though it was only reportable in a single run (Run 1), whereas PFPeA and PFOA were consistently reported across all three runs, averaging 0.17 and 0.15 nmol/h, respectively. Of the remaining PFAS in the biochar tested in this study, PFHpS, NEtFOSE, NMeFOSA, NMeFOSAA, and a suite of perfluoroalkyl carboxylic acids were measured above reporting limits although they were only found in one sample run except PFHpA was observed in two.

Thoma et al. (2022) previously conducted a full‐scale evaluation of the SVCW installation. Good correlation was found between that study and results presented here for the laboratory‐scale system with regard to the type and reduction of PFAS through the system in the solid fraction; no analysis of targeted gaseous PFAS emissions was conducted in the Thoma et al. (2022) evaluation. Dried biosolids represented the largest source of PFAS entering the system, and in both studies, PFOA contributed the greatest overall percentage of the PFAS load. Solid phase PFAS removal efficiencies were estimated to be greater than 81.3% to over 99.9% in the full‐scale study for specific compounds, although only one, of eight, biochar samples contained reportable amounts of PFOS and PFBA, and the removal ranges were calculated using the method detection limits (Thoma et al., 2022).

As noted earlier, PFPeA and PFOA were reportable in all biochar samples. The PFPeA results must be viewed with caution because other researchers have identified analytical interferences with PFPeA stemming from non‐PFAS compounds (Bangma et al., 2023). PFPeA and PFBA only have a single mass transition in the targeted PFAS analytical method used, compared to the two mass transitions for the other analytes evaluated. This leads to suspicion around reportable values that must be verified with high resolution mass spectrometry analyses, not included in this research.

### Thermal oxidizer emissions

Ten PFAS were observed at reportable levels in the flue gas including PFOA, which may not be surprising given its dominance in the dried biosolids. And the reportable levels of PFOA in the combustion air may be contributing to that escaping the thermal oxidizer. However, the combustion air PFOA should be viewed with skepticism. Levels of PFOA were found in the field blank and proof blank “back half” fraction of the OTM‐45 sampling train as well in the field blank “breakthrough” fraction, though the latter two data points were qualified as being an approximate value (J per Tables S9 and S11, reported below the lowest calibration point). The PFOA found in the combustion air was unexpected and could point to XAD cartridge contamination like that found in the back half and breakthrough fractions of the OTM‐45 train. The XAD media check analyses for the “back half” and “breakthrough” analyses did not contain reportable PFOA, implying the sample contamination would have occurred in the field handling or connecting glassware. Winchell et al. (2024) observed similar potential combustion air sample contamination when sampling wastewater sludge incinerators.

The fingerprint of short chain PFAS in the thermal oxidizer flue gas is like that found by Winchell et al. (2024) with respect to PFAS in a multiple hearth furnace flue gas, again suggesting that longer chain PFAS in the wastewater solids feedstock are at least partially thermally degraded as observed in other research applications (Alinezhad et al., 2023; Feng et al., 2015; Singh et al., 2019). Short‐chain PFAS also represented the majority of PFAS in pyrolysis flue gas (post off‐gas combustion) of a unit treating wastewater solids (Sørmo et al., 2023). In Sørmo et al. (2023), perfluoroalkyl carboxylic acids comprised a significant fraction of the short‐chain concentration, leading to similar hypotheses regarding partial thermal degradation or conversion. As with this study, Sørmo et al. (2023) also detected PFBA in the flue gas at high percentages although PFBA was not detected in the pyrolysis feedstock.

A similar study conducted at ICFAR using a laboratory‐scale pyrolysis system reported much higher PFAS in the off‐gas after both low temperature (500°C) and higher temperature pyrolysis (700°C), with 72.9 wt% of the total initial PFAS mass in the biosolids being present in off‐gas after low temperature treatment (Bamdad et al., 2022). The same researchers also found that in higher temperature pyrolysis, an increase in longer chain PFAS was noted in the off‐gas (most significantly PFNA), suggesting their increased presence is due to either reactions of short‐chain PFAS or oxidation of PFAS precursors that generate longer chain compounds. At an initial review, this is in opposition to the relative contribution gas phase emissions and type of PFAS found in the results of this study, which is largely attributable to the fact that the Bamdad et al. (2022) study did not subject the pyrolysis off‐gas to thermal oxidation. However, the ostensible formation of longer chain PFAS in pyrolysis off‐gas may further elucidate the preponderance of short‐chain PFAS in the thermal oxidizer flue gas observed in this study. It is possible that the thermal oxidizer is partially degrading recombinant PFAS in the pyrolysis off‐gas. These studies taken together underpin both the degree to which subsequent thermal treatment of the pyrolysis off‐gas can significantly reduce atmospheric emissions, as well as the need for further research into PFAS characterization and possible transformation in the gas phase of thermal processes.

PFAS removal performance of the laboratory‐scale pyrolysis and thermal oxidizer system exceeded performance of a wastewater sludge incinerator evaluated by Winchell et al. (2024). In that research, the fate of PFAS through two full‐scale wastewater sludge incinerators, one multiple hearth and one fluidized bed, was documented. The wastewater processed by these incinerators contained several of the same PFAS as reported in the dried biosolids for this study. In the multiple hearth incinerator, 95% of the molar mass of PFAS in the wastewater sludge was removed prior to discharging to the atmosphere with the flue gas. The fluidized bed flue gas samples incurred contamination, and a similar performance assessment was not possible. Comparably, the pyrolysis and thermal oxidizer system removed 99.4% of the molar mass of PFAS in the dried biosolids. Perhaps, the staged thermal treatment with separate pyrolysis and thermal oxidation facilitates the higher PFAS treatment efficiency, similarly to other common pollutants (Winchell et al., 2022b). Or it may be possible that the higher temperature in the thermal oxidizer following pyrolysis, roughly 1000°C with a 2 s residence time, compared to the multiple hearth with less than 700°C and 4–5 s of residence time in the exhaust hearth acting as a thermal oxidizer, improved the PFAS treatment efficiency. While differences in the experimental programs are challenging to compare, results suggest that pyrolysis coupled with thermal oxidation could provide similar treatment to incineration and warrants further investigation.

The performance of the pyrolysis and thermal oxidizer system is promising and can be expected to improve when additional air pollution controls, aimed at other pollutants, are installed. The system operating at SVCW, previously evaluated by Thoma et al. (2022), includes a wet scrubber and carbon adsorption systems. The wet scrubber controls particulate matter and acid gasses, while the carbon system removes mercury and organics like dioxins and furans (see Niessen, 2002, for general information on these technologies). Particulate removal can reduce PFAS emissions. In theory, some fraction of the “front half” results representing material captured on the filter of the modified OTM‐45 train (Table 4) will be removed from the flue gas. Also, while not proven in flue gas applications, use of carbon‐based pollution control systems could reduce PFAS emissions (Winchell, Ross, et al., 2021) based on their known sorption characteristics that drive use in drinking water PFAS treatment. Notably, these additional air pollution control technologies do not destroy PFAS and if the contaminated residuals are a concern additional treatment thereof will be necessary.

## CONCLUSIONS AND RESEARCH NEEDS

This study documented the presence of targeted PFAS in all inputs and outputs of a pyrolysis system coupled with a thermal oxidizer. An extensive sampling and analytical program provided data that supported an evaluation of PFAS through the process. Across the system, an average 99.4% PFAS (molar) removal was observed. In the solid phase, the dried biosolids PFAS content was reduced 99.0–99.9% after treatment to produce biochar. The flue gas contained reportable PFAS but less than 0.200% of that fed to the system in the dried biosolids.

While emerging evidence points to pyrolysis and other thermal treatment processes for PFAS destruction, further study is needed to ascertain the degree to which these processes transform PFAS into targeted and non‐targeted PFAS. Additionally, it is important to determine which compounds are destroyed or captured by emissions treatment technologies or remain as VFC byproducts. Future research, leveraging TOF analysis across pyrolysis and thermal oxidation systems with sampling downstream of the pyrolysis unit prior to emissions treatment complemented by recently published analyses like OTM‐50 (USEPA, 2024b) for VFC characterization, will allow a more robust analysis of PFAS transformation and degradation.

Based on the findings of this research, the authors are developing a robust experimental program, like that presented in Winchell et al. (2024), to evaluate the fate of PFAS through both the larger pyrolysis/thermal oxidizer system at SVCW and the laboratory‐scale system hosted by ICFAR using the same feedstock and operating conditions. If performance of the larger system can be validated in the laboratory, researchers can proceed with greater confidence that more economical experiments in the laboratory translate to larger scale systems. This will provide the wastewater industry with a useful tool in combatting the challenges posed by PFAS.

## AUTHOR CONTRIBUTIONS

**Lloyd J. Winchell:** Conceptualization; investigation; funding acquisition; methodology; validation; visualization; writing—review and editing; formal analysis; project administration; data curation; supervision; resources. **Joshua Cullen:** Investigation; methodology; writing—original draft. **John J. Ross:** Conceptualization; investigation; funding acquisition; methodology; writing—review and editing; formal analysis. **Alex Seidel:** Formal analysis; data curation; writing—original draft. **Mary Lou Romero:** Writing—original draft. **Farokh Kakar:** Formal analysis; data curation. **Embrey Bronstad:** Writing—original draft; formal analysis. **Martha J. M. Wells:** Conceptualization; investigation; funding acquisition; methodology; validation; visualization; formal analysis; data curation; supervision. **Naomi B. Klinghoffer:** Writing—review and editing; supervision; validation; methodology. **Franco Berruti:** Supervision; writing—review and editing; conceptualization; methodology; validation. **Alexandre Miot:** Writing—review and editing. **Katherine Y. Bell:** Conceptualization; investigation; funding acquisition; writing—review and editing; methodology; validation; visualization; formal analysis; supervision; data curation.

## Supporting information


**Figure S1.** Lab‐scale pyrolysis reactor at ICFAR.
**Figure S2.** Lab‐scale thermal oxidizer situated next to the pyrolysis reactor.
**Figure S3.** Method OTM‐45 sampling train analytical fractions.
**Figure S4.** Laboratory‐Scale Flue Gas Sampling System.
**Figure S5.** Combustion air sampling scheme.
**Table S1.** Pyrolysis Samples Collected.
**Table S2.** Targeted Polar PFAS Analytes.
**Table S3.** Analytical Material and Instrument Commercial Information.
**Table S4.** Judgement Criteria on Data Usability.
**Table S5.** Dried Biosolids (Sample Point 1) targeted liquid or gas chromatographic–mass spectrometric and general chemistry results (Matrix: Solid). Units: ng/g.
**Table S6** combustion air (Sample Point 2) targeted liquid or gas chromatographic–mass spectrometric results and reporting limits (RL) (Matrix: Air). Units: ng/sample.
**Table S7.** Biochar (Sample Point 3) targeted liquid or gas chromatographic–mass spectrometric and general chemistry results (Matrix: Solid). Units: ng/g.
**Table S8** Flue gas (Sample Point 4 Front Half) targeted liquid or gas chromatographic–mass spectrometric results compared to quality control results (Matrix: Flue Gas). The Field Blank Train (FBT) uses glassware previously used at the current site from a completed run. The Proof Blank Train (PBT) uses glassware before it has been used for sampling. Complete description of FBT and PBT in the OTM‐45 method. Units: ng/sample.
**Table S9** Flue gas (Sample Point 4 Back Half) targeted liquid or gas chromatographic–mass spectrometric results compared to quality control results (Matrix: Flue Gas). The Field Blank Train (FBT) uses glassware previously used at the current site from a completed run. The Proof Blank Train (PBT) uses glassware before it has been used for sampling. Complete description of FBT and PBT in the OTM‐45 method. Units: ng/sample.
**Table S10** Flue gas (Sample Point 4 Impinger Condensate) targeted liquid or gas chromatographic–mass spectrometric results compared to quality control results (Matrix: Flue Gas). The Field Blank Train (FBT) uses glassware previously used at the current site from a completed run. The Proof Blank Train (PBT) uses glassware before it has been used for sampling. Complete description of FBT and PBT in the OTM‐45 method. Units: ng/sample.
**Table S11** Flue gas (Sample Point 4 Breakthrough XAD resin) targeted liquid or gas chromatographic–mass spectrometric results compared to quality control results (Matrix: Flue Gas). The Field Blank Train (FBT) uses glassware previously used at the current site from a completed run. The Proof Blank Train (PBT) uses glassware before it has been used for sampling. Complete description of FBT and PBT in the OTM‐45 method. Units: ng/sample.
**Table S12** Flue gas (Sample Point 4) targeted liquid or gas chromatographic–mass spectrometric reporting limits (RL) (Matrix: Flue Gas). Units: ng/sample.
**Table S13** Flue gas (Sample Point 4) targeted liquid or gas chromatographic–mass spectrometric quality control (QC) reporting limits (RL) (Matrix: Flue Gas). Units: ng/sample.

## Data Availability

The data that support the findings of this study are available from the corresponding author upon reasonable request.
