# Supplementary material for: Fate of biosolids‐bound PFAS through pyrolysis coupled with thermal oxidation for air emissions control
Source: Water Environ Res. 2024 Nov 12;96(11):e11149. doi: 10.1002/wer.11149 (PMC11578938; doi:10.1002/wer.11149)
Supplement: Supplementary file 1 — Figure S1. Lab‐scale pyrolysis reactor at ICFAR. Figure S2. Lab‐scale thermal oxidizer situated next to the pyrolysis reactor. Figure S3. Method OTM‐45 sampling train analytical fractions. Figure S4. Laboratory‐Scale Flue Gas Sampling System. Figure S5. Combustion air sampling scheme. Table S1. Pyrolysis Samples Collected. Table S2. Targeted Polar PFAS Analytes. Table S3. Analytical Material and Instrument Commercial Information. Table S4. Judgement Criteria on Data Usability. Table S5. Dried Biosolids (Sample Point 1) targeted liquid or gas chromatographic–mass spectrometric and general chemistry results (Matrix: Solid). Units: ng/g. Table S6 combustion air (Sample Point 2) targeted liquid or gas chromatographic–mass spectrometric results and reporting limits (RL) (Matrix: Air). Units: ng/sample. Table S7. Biochar (Sample Point 3) targeted liquid or gas chromatographic–mass spectrometric and general chemistry results (Matrix: Solid). Units: ng/g. Table S8 Flue gas (Sample Point 4 Front Half) targeted liquid or gas chromatographic–mass spectrometric results compared to quality control results (Matrix: Flue Gas). The Field Blank Train (FBT) uses glassware previously used at the current site from a completed run. The Proof Blank Train (PBT) uses glassware before it has been used for sampling. Complete description of FBT and PBT in the OTM‐45 method. Units: ng/sample. Table S9 Flue gas (Sample Point 4 Back Half) targeted liquid or gas chromatographic–mass spectrometric results compared to quality control results (Matrix: Flue Gas). The Field Blank Train (FBT) uses glassware previously used at the current site from a completed run. The Proof Blank Train (PBT) uses glassware before it has been used for sampling. Complete description of FBT and PBT in the OTM‐45 method. Units: ng/sample. Table S10 Flue gas (Sample Point 4 Impinger Condensate) targeted liquid or gas chromatographic–mass spectrometric results compared to quality control results (Matrix: Flue Gas). The Field [file WER-96-e11149-s001.pdf]

Supporting Information: Fate of Biosolids-Bound PFAS Through Pyrolysis Coupled with Thermal Oxidation for Air Emissions Control

Lloyd J. Winchell[1], Joshua Cullen[2,] John J. Ross[1], Alex Seidel[1], Mary Lou Romero[1], Farokh Kakar[1], Embrey Bronstad [1], Martha J. M. Wells[3], Naomi B. Klinghoffer[2], Franco Berruti[2], Alexandre Miot[4], Katherine Y. Bell[1]

[1] Brown and Caldwell, Walnut Creek, California, USA, [2] Department of Chemical and Biochemical Engineering, Institute for Chemicals and Fuels from Alternative Resources (ICFAR), Western University, London, Ontario, Canada, [3]EnviroChem Services, Cookeville, Tennessee, USA, [4] Silicon Valley Clean Water, Redwood City, CA, USA

Table of Contents

Supporting Information: Fate of Biosolids-Bound PFAS Through Pyrolysis Coupled with Thermal Oxidation for Air Emissions Control 1

S1: Sample Site Details 2

S2. Sampling Locations 5

S3. Sampling and Analytical Details 5

Solid-Phase Samples 6

Gas-Phase Samples 6

S3.1 Thermal Oxidizer Flue Gas 6

S3.2 Combustion Air 8

S3.3 Sampling Event Details 9

S3.4 Targeted Polar Analytes 10

S3.5 Solids Characteristics 13

S3.6 Data Quality Standards 14

S4. Results 16

References 25

S1: Sample Site Details

In 2021, BC commissioned the installation of a solids pyrolysis and thermal oxidizer laboratory reactor system at Institute for Chemicals and Fuels from Alternative Resources (ICFAR). ICFAR built the system with the ability to pyrolyze a variety of feedstocks, including wastewater solids over a range of operating conditions and process the resulting off-gas stream with a condenser assembly to produce oil or with a thermal oxidizer for emissions control and heat production.

Figure S-1 provides a photo of the pyrolysis reactor system. The system consists of six sections including a feeding system, a pyrolysis reactor, a thermal oxidizer, a char extractor, a condensing unit, and a gas extractor exhaust line. A batch of dried biosolid feedstock is loaded into the hopper located atop the motorized screw feeder. After loading, the tank is sealed and purged with nitrogen, safeguarding the system from oxygen contamination during the initial setup. The pyrolysis reactor is a vertical cylindrical tank with an internal height and diameter of 18.5 cm by 13 cm. It is heated by a 220 V and 2000 W band heater. The feed is directed into the pyrolysis reactor which contains a vertical mechanical mixer that blends hot char with the oncoming feed. The produced char is removed incrementally by the char extractor located at the bottom of the reactor. The char is collected into a separate storage vessel which promotes cooling before collection. Two filters with fine mesh wiring cover the two exit lines at the top of the reactor. These filters contain the solid materials while allowing the condensable vapors and non-condensable gases to pass downstream. The gases and vapors that are produced within the pyrolysis reactor are sent to the bottom of the thermal oxidizer through an insulated tube. The secondary reactor is a vertical 316 stainless steel tube measuring 129 cm by 5 cm in height and internal diameter and encompassed by a vertical 4-zone heater. Each of the 4 zones of the thermal oxidizer require 220 V and 1800 W. At the entrance of the thermal oxidizer, dried air is introduced to facilitate combustion. The pyrolysis off-gases are fed to the center of the reactor via an inner tube while the air is pre-heated within the first half of the main reactor tube. The two streams are then mixed at the center of the reactor.

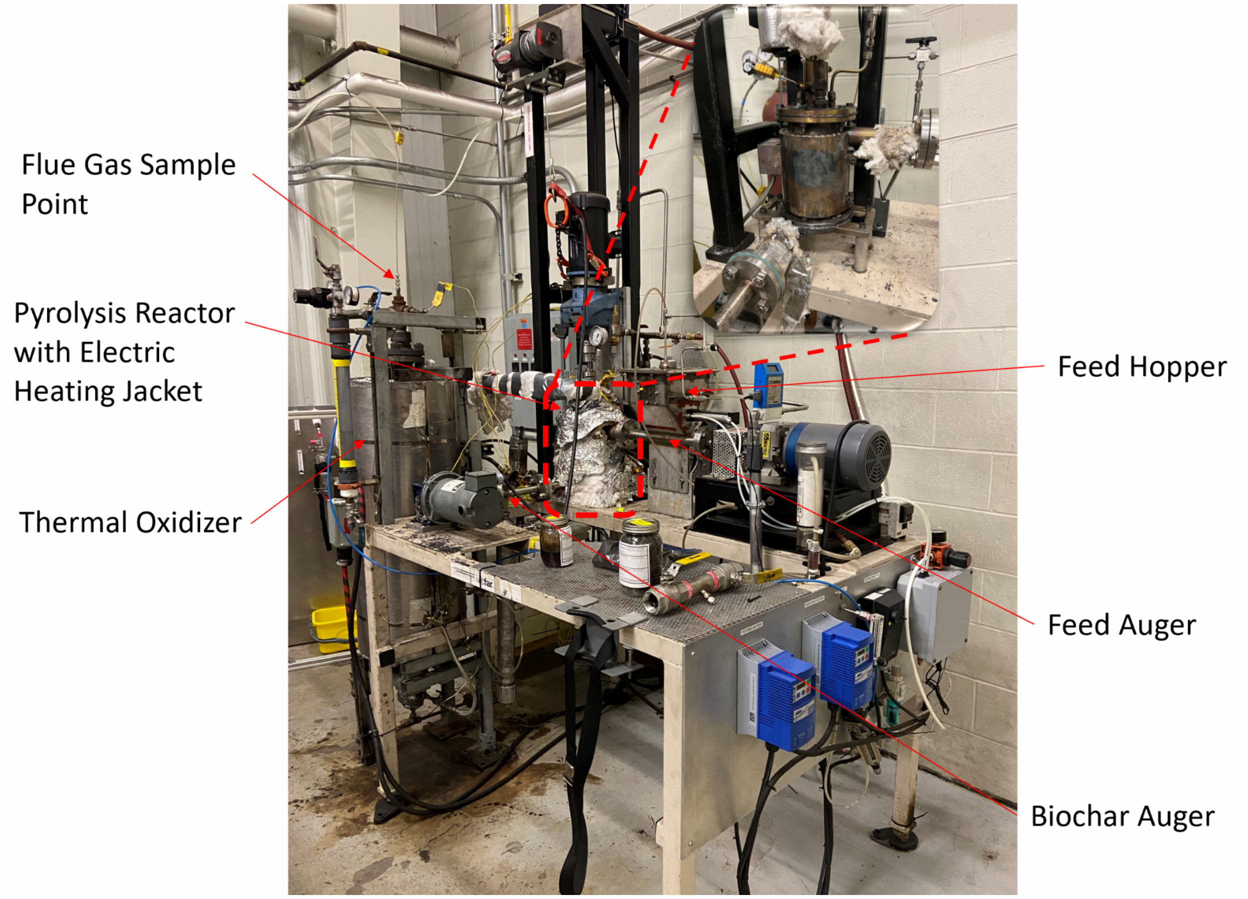

Figure S-1. Lab-scale pyrolysis reactor at ICFAR.

The off-gases produced within the pyrolysis reactor are sent to the bottom of the thermal oxidizer (shown in Figure S-2) through an insulated tube. As the hot flue gases exit the thermal oxidizer, a portion was collected for PFAS. From the flue gas sample collection point, the remaining flue gas was passed through a series of condensers allowing for the condensation of water, oils, and tar produced from pyrolysis treatment. At the end of the exhaust line from the thermal oxidizer, there was a gas extractor to drive the flow of the gases through the system.

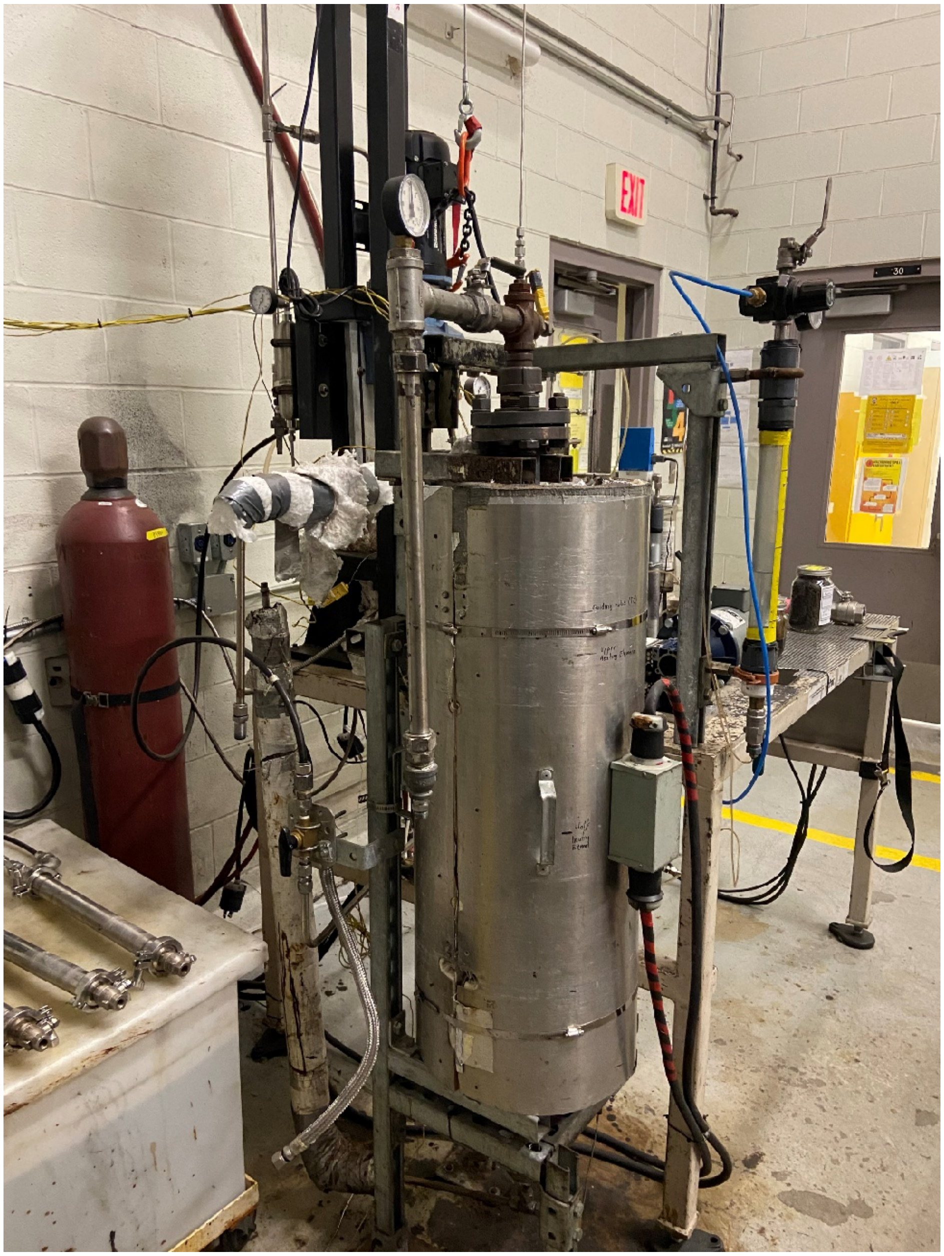

Figure S-2. Lab-scale thermal oxidizer situated next to the pyrolysis reactor.

The laboratory reactor test event consisted of three runs. For each of the three runs, 2 kilograms of thoroughly mixed dried biosolid feedstock was loaded into the hopper. The solids were fed to the pyrolysis reactor on a continuous basis from the feed hopper. A feed auger, with inlet and outlet valves and a nitrogen gas sparging port to remove oxygen after initial setup, was used to continuously introduce dried product into the system. The residence time was calculated by dividing the reactor bed volume by the feed and discharge rate, which was determined considering the mass reduction occurring within the reactor. The pyrolysis reactor was brought to operating temperature prior to introduction of the dried solids and temperature was maintained by automatic modulation of the electrical conduction band heater. A flowmeter measured the exhaust gas flow rate in the exhaust line from the thermal oxidizer. ICFAR was able to use the flowmeter at the very beginning of the runs but had difficulty keeping

moisture from building up in the meter, which resulted in an inability to continuously measure the flow rate. Based on the readings from this meter and the meter from OTM-45, an average exhaust flow rate for each of the runs was calculated. Also, the pyrolysis gas and vapor mass flow rates were based off the feed rate of the biosolids, and outtake of the char. Table 1 provides an overview of the operating conditions for the laboratory system, intended to mimic those of the full-scale system; lambdas (stoichiometric oxygen ratio of available versus combustion requirements) were calculated based on an elemental balance conducted previously using elemental analysis for the solids. Biosolids were used in a separate pyrolysis run at 600°C and a mass and elemental distribution of the carbon, hydrogen, nitrogen, sulfur, and oxygen, and ash content in the biosolids was determined. The distribution in conjunction with the yield and elemental makeup of the biochar were then used to calculate the yield and remaining elemental mass flows of the vapors and gases (as a combined stream). This was calculated as a mass difference between the biosolids and the biochar. Based on these mass flows and the air input into the thermal oxidizer (15 L/min), lambda and excess air were determined. The target lambda was 1.3 for all the runs based on a consistent feeding rate but because of the plugging in the feeding line, higher lambdas resulted.

Table 2 summarizes three experimental runs, detailing the feed rate of dried biosolids, biochar yield, off-gas rate, and air supply to the thermal oxidizer.

S2. Sampling Locations

To properly track PFAS through the pyrolysis system the study sampled two phases—gases and solids. Water will be part of these samples but not as discrete liquid samples except in the flue gas sampling train. PFAS exist in volatile, semi-volatile, and non-volatile forms. This research targeted the semi-volatile and non-volatile forms using the sampling procedure discussed later. Figure 1 shows the correlated sample points for the laboratory-scale system.

Sample point 1: The dried biosolids material from SVCW was sampled from each experimental run. Typically, the material is 80 percent total solids (TS).

Sample Point 2: The pyrolysis process intentionally leaves a portion of the combustible matter present in the biosolids as a solid residual along with the non-combustible fraction, known as biochar. ICFAR collected a sample from each experimental run. The biochar is a single-phase matrix with no retained water.

Sample Point 3: Combustion processes require oxygen, usually supplied from an air stream. Combustion air samples in concert with each flue gas emission samples were collected.

Sample Point 4: Samples of flue gas after the thermal oxidizer.

S3. Sampling and Analytical Details

Eurofins Test America (ETA), Knoxville, TN, USA and Lancaster, PA, USA provided all bottles for sample collection and storage. ETA also supplied sampling equipment for collecting and compositing samples

and Amberlite® XAD resin cartridges and particulate filters for gas phase sampling. ORTECH Consulting Inc (ORTECH), Mississauga, Ontario, Canada provided all labware and equipment for taking gas-phase samples from combustion air and flue gas.

Solid-Phase Samples

Samples of dried biosolids and biochar were collected by taking representative samples of the material fed to, or resulting from, the process run.

Dried biosolids and biochar contain no filterable liquid phase. These samples were processed in the same manner: raw samples underwent a polar extraction using a 0.4% potassium hydroxide (KOH) and methanol solution subjected to shaking and then sonication with a final pH adjustment.

Polar extract cleaned with solid-phase extraction (SPE) using a weak anion exchange (WAX) resin, analyte elution using 0.3% NH_4OH and methanol solution.

Gas-Phase Samples

Gas phase sampling included flue gas and combustion air conducted by ORTECH. Flue gas sampling followed "Other Test Method 45 (OTM-45) Measurement of Selected Per- and Polyfluorinated Alkyl Substances from Stationary Sources" for semi-volatile and non-volatile compounds (USEPA, 2021). Combustion air was sampled per the procedure described in Section S3.2. All sampling was conducted in triplicate.

S3.1 Thermal Oxidizer Flue Gas

The flue gas sample apparatus was assembled (Figure S-3) according to the "Other Test Method 45 (OTM-45) Measurement of Selected Per- and Polyfluorinated Alkyl Substances from Stationary Sources" (USEPA, 2021) by ORT. Four analytical samples are collected by the Method OTM-45 sampling train:

1. Particulate filter and front half of the filter holder, nozzle, and probe solvent rinses;
2. XAD-2 resin trap and back half of the filter holder, coil condenser and connecting glassware solvent rinses;
3. Condensate, impinger contents and their related glassware rinses; and
4. Breakthrough XAD-2 module.

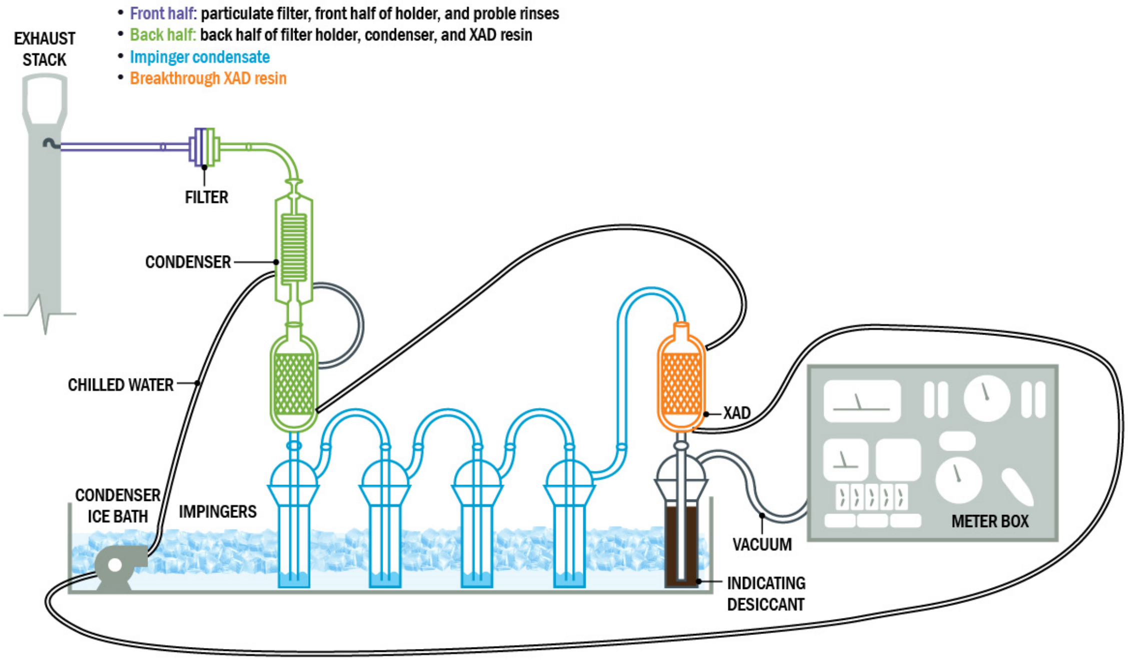

Figure S-3. Method OTM-45 sampling train analytical fractions.

The laboratory-scale flue gas sampling followed OTM-45 where possible by ORTECH with the following modifications.

- OTM-45 is an Isokinetic method and due to the size of the exhaust and the volume of gas exhausted it would not be possible to sample isokinetic, Instead, a constant rate sample was collected.
- Smaller impingers were used due to the relatively low sample rate. The low sampling rate ensured contaminating ambient air was not pulled into the sampling train.

The sampling apparatus conformed to SW-846 Method 0030 (Volatile Organic Sampling Train) to accommodate the small process lines and flow rates except the larger XAD cartridges associated with the full size OTM-45 were used. Figure S-4 shows the sampling system which includes the same main components as OTM-45. The seven discrete sampling fractions described in OTM-45 were collected and delivered to ETA. These include the filter and rinses of the front half, the back half XAD and rinses, impinger liquid and rinses, and breakthrough XAD. The related rinses are combined with the other portions of each of the four fractions shown in Figure S-3 to yield the final reported value.

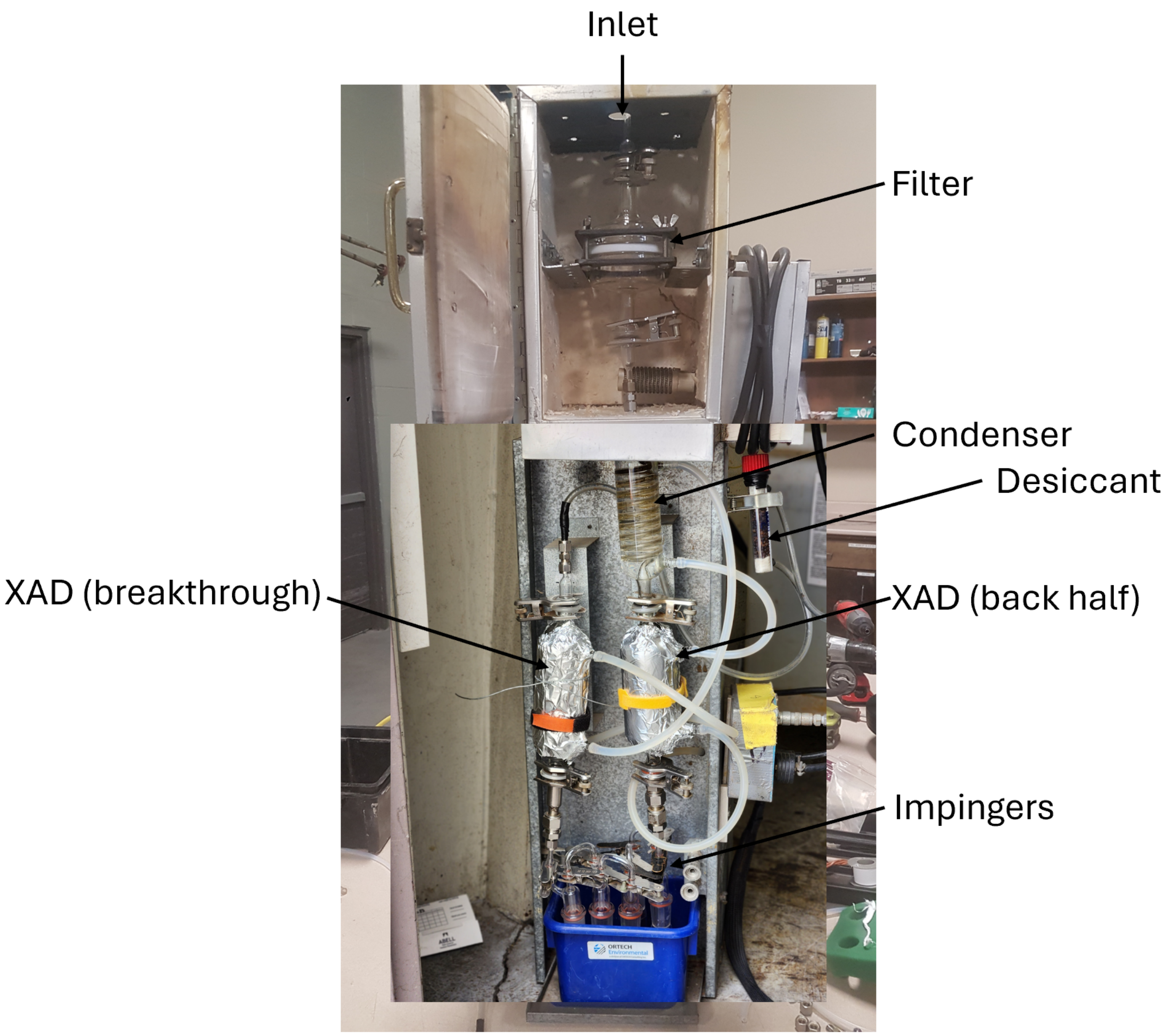

Figure S-4. Laboratory-Scale Flue Gas Sampling System.

ORTECH provided a sample pump, tubing, and XAD cartridge holder to collect combustion air during the same period as the flue gas sampling. A split of the flue gas was collected at a rate of approximately 3 L/min (dry) measured by the dry gas meter for a total of 907.00 L, 947.70 L, and 539.46 L for Runs 1, 2, and 3, respectively. This method approximates the Modified Method OTM-45 approach. ORTECH relinquished each XAD cartridge to ETA after sample collection. Samples were stored at 4°C.

S3.2 Combustion Air

The sample train at the combustion air location consisted of a XAD-2 resin trap followed by a condensate knockout impinger as shown in Figure S-5. The samples were collected at a constant rate, approximately 4 L/min dry, concurrently with the flue gas samples for a total of 1,137.16 L, 1,217.65 L, and 691.51 L for Runs 1, 2 , and 3, respectively. Run 3 volume was less than the other runs due to issues maintaining a

temperature of 1000°C. The heating element temperature began to drop from over 1000°C to around 850°C, and the team was unable to fix the issue to continue the run.

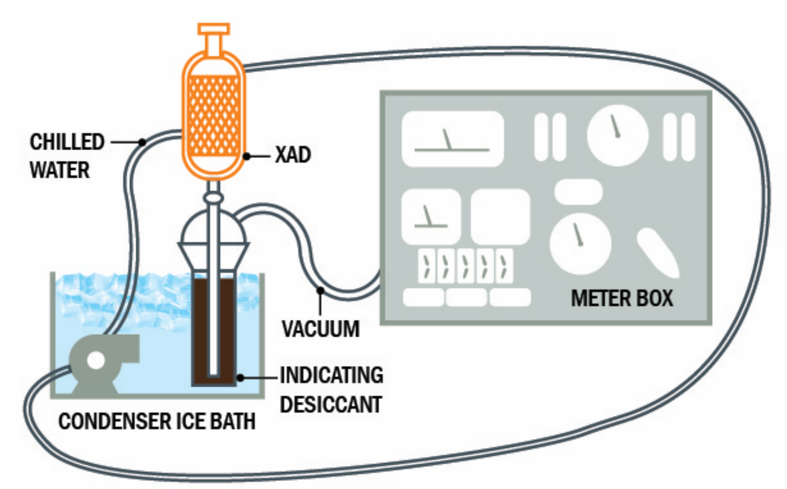

Figure S-5. Combustion air sampling scheme.

S3.3 Sampling Event Details

Sampling took place on May 30, June 1, and June 2 of 2023. The three sampling runs were completed per the following:

Run 1 — 5 hours in duration on May 30, 2023.

Run 2 — 5 hours in duration on June 1, 2023.

Run 3 — 2.5 hours in duration on June 2, 2023. Run 3 was shorter than the previous 2 days due to issues maintaining a temperature of 1000°C. As previously noted, the heating element temperature began to drop from over 1000°C to around 850°C, and the team was unable to fix the issue to continue the run.

Table S-1 identifies the samples collected during the sampling event.

Table S-1. Pyrolysis Samples Collected.			
**Stream**	**Sample Point (Figure-1)**	**Collection Type**	**Samples**
**Dried biosolids**	2	Grabs	3 + 1 field duplicate
**Biochar**	3	Grabs	3
**Combustion air**	4	Custom	3
**Flue gas**	5	Modified Method OTM-45	3 particulate filters<br>3 front half methanol/NH4OH rinse<br>3 front XAD cartridges<br>3 back half methanol/NH4OH rinse<br>3 impinger condensate<br>3 impinger methanol/NH4OH rinse<br>3 breakthrough XAD cartridges<br>1 particulate filter field blank<br>1 front half methanol/NH4OH field blank<br>1 front XAD cartridge field blank<br>1 back half methanol/NH4OH field blank<br>1 impinger condensate field blank<br>1 impinger methanol/NH4OH field blank<br>1 breakthrough XAD cartridge field blank<br>1 particulate filter proof blank<br>1 front half methanol/NH4OH proof blank<br>1 front XAD cartridge proof blank<br>1 back half methanol/NH4OH proof blank<br>1 impinger condensate proof blank<br>1 impinger methanol/NH4OH proof blank<br>1 breakthrough XAD cartridge proof blank

S3.4 Targeted Polar Analytes

Quantitative targeted analyses utilize known reference standards, based on standardized regulatory methods and extended methods established by ETA. Targeted methods are based on low resolution mass spectrometric detection (LRMS) by either liquid or gas chromatography with tandem mass spectrometry (LC-MS/MS or GC-MS/MS). Targeted methods generate quantitative data.

The USEPA currently has standardized two methods (537.1 and 533, USEPA, 2019) for quantifying PFAS, mainly polar compounds, in drinking water. During the project, USEPA Method 1633 was released in draft and then finalized in January 2024 (USEPA, 2024) after samples from this project were analyzed.

Commercial laboratories also use the USEPA methods with modifications to analyze other matrices such as non-potable water, soils/sediments, and gas phase samples. Samples are prepared using a SPE procedure. Together, these methods cover 29 PFAS. Many commercial laboratories offer an expanded list of PFAS, made possible from proprietary standards. Non-polar compounds do not have a standardized method yet. Again, some commercial laboratories offer proprietary gas chromatography (GC-MS/MS) methods to measure these non-polar compounds. ETA analyzed each sample point for the targeted list PFAS in Table S-2, though some variation in the reported analytes by sample are reflected in the results tables. ETA reported each analyte as a concentration from the liquid or solid phase samples (e.g., nano-grams per liter or gram [ng/L or ng/g]) and on a sample basis for some of the gas phase sample fractions (e.g., ng/sample).

Table S-2. Targeted Polar PFAS Analytes

Family	Full Name	Acronym	CAS	Molecular Weight (g/mol) of acid form
Perfluoroalkane carboxylic acids (PFCA)	Perfluorobutanoic acid	PFBA	375-22-4	214.04
	Perfluoropentanoic acid	PFPeA	2706-90-3	264.05
	Perfluorohexanoic acid	PFHxA	307-24-4	314.05
	Perfluoroheptanoic acid	PFHpA	375-85-9	364.06
	Perfluorooctanoic acid	PFOA	335-67-1	414.07
	Perfluorononanoic acid	PFNA	375-95-1	464.08
	Perfluorodecanoic acid	PFDA	335-76-2	514.08
	Perfluoroundecanoic acid	PFUnA	2058-94-8	564.09
	Perfluorododecanoic acid	PFDoA	307-55-1	614.10
	Perfluorotridecanoic acid	PFTrDA	72629-94-8	664.11
	Perfluorotetradecanoic acid	PFTeDA	376-06-7	714.11
	Perfluoro-n-hexadecanoic acid	PFHxDA	67905-19-5	814.13
	Perfluoro-n-octadecanoic acid	PFODA	16517-11-6	914.14
Perfluoroalkane sulfonic acids (PFSA)	Perfluorobutanesulfonic acid	PFBS	375-73-5	300.10
	Perfluoropentanesulfonic acid	PFPeS	2706-91-4	350.11
	Perfluorohexanesulfonic acid	PFHxS	355-46-4	400.12
	Perfluoroheptanesulfonic acid	PFHpS	375-92-8	450.12
	Perfluorooctanesulfonic acid	PFOS	1763-23-1	500.13
	Perfluorononanesulfonic acid	PFNS	68259-12-1	550.14

	Perfluorodecanesulfonic acid	PFDS	335-77-3	600.15
	Perfluorododecanesulfonic acid	PFDoS	79780-39-5	700.16
	Perfluoro-4-ethylcyclohexanesulfonic acid	PFECHS	646-83-3	462.13
**Per/poly-fluorinated ether carboxylic acids (PFECA)**	4,8-dioxa-3H-perfluorononanoic acid	ADONA	919005-14-4	378.07
	Hexafluoropropylene oxide dimer acid	HFPO-DA (GenX)	13252-13-6	330.05
	Nonafluoro-3,6-dioxaheptanoic acid	NFDHA	151772-58-6	296.04
**Per/poly-fluorinated ether sulfonic acids (PFESA)**	9-chlorohexadecafluoro-3-oxanonane-1-sulfonic acid	9Cl-PF3ONS (F53B major)	756426-58-1	532.58
	11-chloroeicosafluoro-3-oxaundecane-1-sulfonic acid	11Cl-PF3OUdS (F53B minor)	763051-92-9	632.60
	Perfluoro-(2-ethoxyethane)-sulfonic acid	PFEESA	113507-82-7	316.10
	Perfluoro-3-methoxypropanoic acid	PFMPA	377-73-1	230.04
	Perfluoro-4-methoxybutanoic acid	PFMBA	863090-89-5	280.04
**Perfluoroalkane sulfonamide (FASA)**	Perfluorooctanesulfonamide	FOSA	754-91-6	499.15
	N-methylperfluorooctane sulfonamide	NMeFOSA	31506-32-8	513.169
	N-ethylperfluorooctane sulfonamide	NEtFOSA	4151-50-2	527.20
**Perfluoroalkane sulfonamido acetic acid (FASAA)**	N-methylperfluorooctanesulfonamidoacetic acid	NMeFOSAA	2355-31-9	571.21
	N-ethylperfluorooctanesulfonamido acetic acid	NEtFOSAA	2991-50-6	585.24
Perfluoroalkane sulfonamido ethanol (FASE)	2-(N-methylperfluoro-1-octanesulfonamido) ethanol	NMeFOSE	24448-09-7	557.23
	2-(N-ethylperfluoro-1-octanesulfonamido) ethanol	NEtFOSE	1691-99-2	571.25
	3-Perfluoropentylpropanoic acid	5:3 FTCA	914637-49-3	342.11
	6:2 Fluorotelomer carboxylic acid	6:2 FTCA	53826-12-3	378.09

**Fluorotelomer based carboxylic acids (FTCA)**	6:2 Fluorotelomer unsaturated carboxylic acid	6:2 FTUCA	70887-88-6	358.08
	3-Perfluoroheptylpropanoic acid	7:3 FTCA	812-70-4	442.12
	10:2 Fluorotelomer carboxylic acid	10:2 FTCA	53826-13-4	578.12
	8:2 Fluorotelomer carboxylic acid	8:2 FTCA	27854-31-5	478.10
	8:2 Fluorotelomer unsaturated carboxylic acid	8:2 FTUCA	70887-84-2	458.10
	3-Perfluoropropylpropanoic acid	3:3 FTCA	356-02-5	242.09
**Fluorotelomer sulfonic acids (FTSA)**	1H,1H,2H,2H-perfluorohexane sulfonic acid	4:2 FTS	757124-72-4	328.15
	1H,1H,2H,2H-perfluorooctane sulfonic acid	6:2 FTS	27619-97-2	428.17
	1H,1H,2H,2H-perfluorodecane sulfonic acid	8:2 FTS	39108-34-4	528.18
	1H,1H,2H,2H-perfluorododecane sulfonic acid	10:2 FTS	120226-60-0	628.20

ETA used the material, reagents and sorbents, and instruments listed in Table S-3.

Table S-3. Analytical Material and Instrument Commercial Information

**Chemical or Instrument**	Vendor	Geographical Information
Ammonium hydroxide	**ThermoFisher Scientific**	**Fair Lawn, NJ**
Methanol	**ThermoFisher Scientific**	**Fair Lawn, NJ**
Sand	**ThermoFisher Scientific**	**Fair Lawn, NJ**
Potassium hydroxide	**ThermoFisher Scientific**	**Fair Lawn, NJ**
SPE/WAX sorbent	**Phenomenex Strata PFAS 500mg/50mg/6mL Lot: X1118-TB**	**Torrance, CA**
Sodium Hydroxide	**ThermoFisher Scientific**	**Fair Lawn, NJ**
LC-MS/MS	**SCIEX**	**Framingham, MA**

S3.5 Solids Characteristics

Total solids and volatile solids analysis were conducted by ETA, Pittsburgh, PA using Standard Method 2540G.

S3.6 Data Quality Standards

Project analytical data were assessed for data quality using several means: (1) the Quality Assurance/Quality Control (QA/QC) data provided by the analytical laboratory partners, (2) the Brown and Caldwell data verification and validation guidelines for reporting general chemistry parameters; (3) guidance documents from USEPA (2002) and Interstate Technology & Regulatory Council (ITRC, 2022a and 2022b); (4) though not yet promulgated at the time, guidance from USEPA Draft Method 1633 (USEPA, 2022b) was considered; and (5) the experience/expertise of the project research team.

Data qualifiers were interpreted by the team to assess data usability. A list of qualifiers and definitions encountered and their interpretation for the determination of data usability in this project is presented in Table S-4.

Among the most important data quality parameters is the sensitivity of the analysis. Numerous terms and acronyms have evolved in the literature to describe “detection” and “quantitation” limits (USEPA, 1995 and ITRC, 2022a). In these data, the terms minimum detection limit (MDL) and reporting limit (RL) are used to report the “detection” and “quantitation” limits, respectively. The MDL is defined as the minimum measured concentration of a substance that can be reported with 99 percent confidence that the measured concentration is distinguishable from method blank results (USEPA, 2017d). The RL is the lowest nonzero calibration point in the calibration curve for each analyte. Because of varying properties between samples, that is, sample size, matrix effects, dilutions made during analysis, the RL can vary from sample to sample and analyte to analyte (ITRC, 2022b). The RL values for each analyte in each sample are provided in the data tables within this SI.

The “J” qualifier represents an estimated analytical result between the MDL and the RL. The project team determined that although the numerical value having a J qualifier as reported by the laboratory was a valid indicator of data “quality,” the J qualifier, represents an estimation outside the calibration curve, and therefore, its data “usability” is unfit. Data having J qualifiers, regardless of other qualifiers listed for the sample, were not reported numerically but were reported as “J.” No numerical values were reported for the data qualifiers I, CI, *+, and *1; the numerical value was replaced by the data qualifier itself, alerting the data user that the result could not be confirmed.

For the “B” data qualifier associated with the chromatographic quantitative data, no numerical values were reported; the numerical value was replaced by the data qualifier itself, alerting the data user that the result could not be confirmed. Per USEPA Draft Method 1633: Analysis of Per- and Polyfluoroalkyl Substances (PFAS) in Aqueous, Solid, Biosolids, and Tissue Samples by LC-MS/MS. “Results associated with blank contamination for an analyte regulated in a discharge cannot be used to demonstrate regulatory compliance.”

Table S-4. Judgement Criteria on Data Usability		
**Data Qualifiers**	Assessment of Data Usability	Data Reported As
**ND**	Not detected at the MDL.	ND
**J**	Result is less than the RL but greater than or equal to the MDL and the concentration is an approximate value. Qualifier indicates quantitation outside the calibration range. The result is estimated/uncertain with indeterminate bias. Data determined to be unusable.	J (no numerical value reported)
**I**	Value is estimated maximum possible concentration (EMPC). Interference. Data determined to be unusable.	I (no numerical value reported)
**CI**	The peak identified by the data system exhibited chromatographic interference that could not be resolved. There is reason to suspect there may be a high bias. Data determined to be unusable.	CI (no numerical value reported)
***+**	Lab Control Sample (LCS) and/or Lab Control Sample Duplicate (LCSD) is outside acceptance limits, high biased. Data determined to be unusable.	*+ (no numerical value reported)
***1**	LCS/LCSD Relative Percent Difference (RPD) exceeds control limits. Data determined to be unusable.	*1 (no numerical value reported)
**B**	Compound was found in the blank and sample. Data determined to be unusable.	B (no numerical value reported)
**H**	Sample was prepped or analyzed beyond the specified holding time. Holding times have not been established.	Numerical value reported with H superscript

S4. Results

For the purposes of evaluating the results PFAS were characterized as long or short chain variants. Long-chain PFAS compounds were defined as any PFAS compound with a fully fluorinated chain of eight or more carbons, with exceptions for two PFAS families. Perfluoroalkyl carboxylic acids with chains of seven or more, and perfluoroalkyl sulfonic acids with chains of six or more fully fluorinated carbons were considered long-chain PFAS (Buck et al., 2011). All other PFAS compounds were considered as short-chain.

Table S-5. Dried Biosolids (Sample Point 1) targeted liquid or gas chromatographic–mass spectrometric and general chemistry results (Matrix: Solid). Units: ng/g.

Family	Acronym	Dried Biosolids				Reporting Limits (RL)			
		R1	R2a	R2b	R3	R1	R2a	R2b	R3
	PFBA	B	B	BR	B	0.990	1.98	1.99	2.00
	PFPeA	1.78	2.05	2.33	J	0.990	1.98	1.99	2.00
	PFHxA	12.5	14.4	13.0	13.8	0.990	1.98	1.99	2.00
	PFHpA	3.76	4.60	3.64	4.13	0.990	1.98	1.99	2.00
	PFOA	70.5	73.3	71.0	75.7	0.990	1.98	1.99	2.00
	PFNA	2.58	2.82	2.48	2.63	0.990	1.98	1.99	2.00
	PFDA	8.45	10.2	8.77	9.95	0.990	1.98	1.99	2.00
	PFUnA	1.32	J	J	J	0.990	1.98	1.99	2.00
	PFDoA	R	R	2.85	3.28	0.990	1.98	1.99	2.00
	PFTriA	J*-R	JI*-R	*-	*-	0.990	1.98	1.99	2.00
	PFTeA	R	R	R	R	0.990	1.98	1.99	2.00
	PFHxA	R	R	R	<MDL	0.990	1.98	1.99	2.00
Perfluoroalkyl Carboxylic Acids	PFOA	*+R	*-R	*-R	*+	0.990	1.98	1.99	2.00
	PFBS	I	J	J	JI	0.990	1.98	1.99	2.00
	PFPeS	<MDL	<MDL	<MDL	<MDL	0.990	1.98	1.99	2.00
	PFHxS	I	JI	JI	JI	0.990	1.98	1.99	2.00
	PFHpS	<MDL	<MDL	<MDL	<MDL	0.990	1.98	1.99	2.00
	PFOS	19.6	19.0	16.7	22.1	0.990	1.98	1.99	2.00
	PFNS	<MDL	<MDL	<MDL	<MDL	0.990	1.98	1.99	2.00
	PFDS	*-	J*-	JI*-	J*-	0.990	1.98	1.99	2.00
Perfluoroalkane Sulfonates	PFDoS	*-*1	I*-*1	*-*1	I*-*1	0.990	1.98	1.99	2.00
	FOSA	J	J	J	J	0.990	1.98	1.99	2.00
	NEtFOSA	R	R	R	R	0.990	1.98	1.99	2.00
	NMeFOSA	<MDL	<MDL	<MDL	<MDL	0.990	1.98	1.99	2.00
	NMeFOSAA	15.5	15.8	14.9	15.9	0.990	1.98	1.99	2.00
	NEtFOSAA	13.7	15.2	13.7	15.1	0.990	1.98	1.99	2.00
	NMeFOSE	10.8	NR	NR	NR	0.990	1.98	1.99	2.00
Perfluorosulfonamides	NEtFOSE	R	NR	NR	2.30	0.990	1.98	1.99	2.00
	4:2 FTS	<MDL	<MDL	<MDL	<MDL	0.990	1.98	1.99	2.00
	6:2 FTS	<MDL	<MDL	<MDL	<MDL	0.990	1.98	1.99	2.00
	8:2 FTS	J	J	J	J	0.990	1.98	1.99	2.00
Fluorotelomer Sulfonates	10:2 FTS	J	<MDL	J	J	0.990	1.98	1.99	2.00
	ADONA	<MDL	<MDL	<MDL	<MDL	0.990	1.98	1.99	2.00
Perfluoroether Carboxylic Acids	HFPO-DA	<MDL	<MDL	<MDL	<MDL	0.990	1.98	1.99	2.00
Chlorinated Polyfluoroalkyl Ether	9Cl-PF3ONS	<MDL	<MDL	<MDL	<MDL	0.990	1.98	1.99	2.00
Sulfonic Acids	11Cl-PF3OUdS	*-*1	*-*1	*-*1	*-*1	0.990	1.98	1.99	2.00
General Chemistry									
Total Volatile Solids		66.5	67.4	67.2	67.5	0.500	0.500	0.500	0.500
Solids, total		76.3	73.8	73.6	81.6	0.500	0.500	0.500	0.500

NR: Not Recoverable.

J: Result is less than the RL but greater than or equal to the MDL and the concentration is an approximate value.

B: Compound was found in the blank and sample.

I: Value is EMPC (estimated maximum possible concentration).

*-: LCS and/or LCSD is outside acceptance limits, low biased.

R: Rejected sample

<MDL: Non Detectable

Note that all analytes were subject to H or H3 qualifiers, indicating the sample was prepped or analyzed or received beyond the specific holding time. The sample does not meet regulatory requirements

Table S-6 combustion air (Sample Point 2) targeted liquid or gas chromatographic–mass spectrometric results and reporting limits (RL) (Matrix: Air). Units: ng/sample.

Family	Acronym	Combustion Air			Reporting Limits (RL)		
		R1	R2	R3	R1	R2	R3
	PFBA	CI	CI	CI	10.0	10.0	10.0
	PFPeA	<MDL	<MDL	<MDL	1.00	1.00	1.00
	PFHxA	<MDL	<MDL	<MDL	1.00	1.00	1.00
	PFHpA	<MDL	<MDL	<MDL	3.00	3.00	3.00
	PFOA	3.88	1.28	1.29	1.00	1.00	1.00
	PFNA	<MDL	<MDL	<MDL	1.00	1.00	1.00
	PFDA	<MDL	<MDL	<MDL	1.00	1.00	1.00
	PFUnA	<MDL	<MDL	<MDL	1.00	1.00	1.00
	PFDoA	<MDL	R	R	1.00	1.00	1.00
	PFTrDA	<MDL	R	R	1.00	1.00	1.00
	PFTeA	R	R	R	1.00	1.00	1.00
	PFHxDA	R	R	R	1.00	1.00	1.00
Perfluorocarboxylic Acids (PFCAs)	PFODA	*- R	*-R	*-R	1.00	1.00	1.00
	PFBS	<MDL	<MDL	<MDL	1.00	1.00	1.00
	PFPeS	<MDL	<MDL	<MDL	1.00	1.00	1.00
	PFHxS	<MDL	<MDL	<MDL	1.00	1.00	1.00
	PFHpS	<MDL	<MDL	<MDL	1.00	1.00	1.00
	PFOS	<MDL	JI	<MDL	1.00	1.00	1.00
	PFNS	<MDL	<MDL	<MDL	1.00	1.00	1.00
	PFDS	<MDL	<MDL	<MDL	1.00	1.00	1.00
Perfluorosulfonates (PFSAs)	PFDoS	*-	*-	*-	1.00	1.00	1.00
	FOSA	<MDL	<MDL	<MDL	1.00	1.00	1.00
	NEtFOSA	<MDL	<MDL	<MDL	1.00	1.00	1.00
	NMeFOSA	<MDL	<MDL	<MDL	1.00	1.00	1.00
	NMeFOSAA	<MDL	<MDL	<MDL	1.00	1.00	1.00
	NEtFOSAA	<MDL	<MDL	<MDL	1.00	1.00	1.00
	NMeFOSE	<MDL	<MDL	<MDL	20.0	20.0	20.0
Sulfonamides	NEtFOSE	<MDL	<MDL	<MDL	1.00	1.00	1.00
	4:2 FTS	<MDL	<MDL	<MDL	1.00	1.00	1.00
	6:2 FTS	<MDL	<MDL	<MDL	10.0	10.0	10.0
	8:2 FTS	<MDL	<MDL	<MDL	1.00	1.00	1.00
Fluorotelomers	10:2 FTS	<MDL	R	<MDL	1.00	1.00	1.00
	ADONA	<MDL	<MDL	<MDL	2.00	2.00	2.00
Polyfluoroalkyl Substances	HFPO-DA	<MDL	<MDL	<MDL	20.0	20.0	20.0
	9Cl-PF3ONS	<MDL	<MDL	<MDL	1.00	1.00	1.00
Chlorinated Polyfluoroalkyl Substances	11Cl-PF3OUdS	<MDL	<MDL	<MDL	1.00	1.00	1.00
Fluorotelomer Unsaturated Carboxylic Acids	6:2 FTUCA	<MDL	<MDL	<MDL	1.00	1.00	1.00
	7:3 FTCA	*+	*+	*+	1.00	1.00	1.00
	10:2 FTCA	<MDL	<MDL	<MDL	1.00	1.00	1.00
Fluorotelomer Carboxylic Acids	8:2 FTCA	<MDL	<MDL	<MDL	1.00	1.00	1.00
Ethoxylated Substances	PFEESA	<MDL	<MDL	<MDL	1.00	1.00	1.00
Fluorotelomer Unsaturated Carboxylic Acids	8:2 FTUCA	<MDL	<MDL	<MDL	1.00	1.00	1.00
Methoxylated Substances	PFMPA	<MDL	<MDL	<MDL	1.00	1.00	1.00
Oxahexanoic Substances	PFMBA	<MDL	<MDL	<MDL	1.00	1.00	1.00
	5:3 FTCA	*+	*+	*+	1.00	1.00	1.00
	6:2 FTCA	<MDL	<MDL	<MDL	1.00	1.00	1.00
Fluorotelomer Carboxylic Acids	3:3 FTCA	<MDL	<MDL	<MDL	1.00	1.00	1.00
Ethoxylated Substances	PFECHS	<MDL	<MDL	<MDL	1.00	1.00	1.00
Dioxaheptanoic Substances	NFDHA	<MDL	<MDL	<MDL	1.00	1.00	1.00

J: Result is less than the RL but greater than or equal to the MDL and the concentration is an approximate value.

I: Value is EMPC (estimated maximum possible concentration).

<MDL: Non Detectable

*+: LCS and/or LCSD is outside acceptance limits, high biased.

*-: LCS and/or LCSD is outside acceptance limits, low biased.

Table S-7. Biochar (Sample Point 3) targeted liquid or gas chromatographic–mass spectrometric and general chemistry results (Matrix: Solid). Units: ng/g.

Family	Acronym	Biochar				Reporting Limits (RL)		
		R1	R2	R3		R1	R2	R3
Perfluoroalkyl Carboxylic Acids	PFBA	BR	BCI	B		0.197	0.0986	0.0988
	PFPeA	0.716	0.387	0.170		0.197	0.0986	0.0988
	PFHxA	I	0.474	I		0.197	0.0986	0.0988
	PFHpA	0.205	0.138	J		0.197	0.0986	0.0988
	PFOA	0.953	0.548	0.234		0.197	0.0986	0.0988
	PFNA	J	J	J		0.197	0.0986	0.0988
	PFDA	J	0.122	J		0.197	0.0986	0.0988
	PFUnA	<MDL	<MDL	<MDL		0.197	0.0986	0.0988
	PFDoA	JI	J	JR		0.197	0.0986	0.0988
	PFTriA	*-	*-	*-R		0.197	0.0986	0.0988
	PFTeA	<MDL	R	R		0.197	0.0986	0.0988
	PFHxA	R	<MDL	R		0.197	0.0986	0.0988
	PFOA	*+R	*+	*+R		0.197	0.0986	0.0988
Perfluoroalkane Sulfonates	PFBS	J	<MDL	<MDL		0.197	0.0986	0.0988
	PFPeS	<MDL	<MDL	<MDL		0.197	0.0986	0.0988
	PFHxS	J	<MDL	<MDL		0.197	0.0986	0.0988
	PFHpS	<MDL	<MDL	<MDL		0.197	0.0986	0.0988
	PFOS	2.05	JI	<MDL		0.197	0.0986	0.0988
	PFNS	<MDL	<MDL	<MDL		0.197	0.0986	0.0988
	PFDS	J*-	*-	*-		0.197	0.0986	0.0988
	PFDoS	I*-*1	*-*1	*-*1		0.197	0.0986	0.0988
Perfluorosulfonamides	FOSA	<MDL	<MDL	<MDL		0.197	0.0986	0.0988
	NEtFOSA	<MDL	<MDL	<MDL		0.197	0.0986	0.0988
	NMeFOSA	<MDL	<MDL	<MDL		0.197	0.0986	0.0988
	NMeFOSAA	0.215	<MDL	<MDL		0.197	0.0986	0.0988
	NEtFOSAA	J	<MDL	<MDL		0.197	0.0986	0.0988
	NMeFOSE	0.522	R	JR		0.197	0.0986	0.0988
	NEtFOSE	0.206	R	R		0.197	0.0986	0.0988
Fluorotelomer Sulfonates	4:2 FTS	<MDL	<MDL	<MDL		0.197	0.0986	0.0988
	6:2 FTS	<MDL	<MDL	<MDL		0.197	0.0986	0.0988
	8:2 FTS	J	<MDL	<MDL		0.197	0.0986	0.0988
	10:2 FTS	<MDL	<MDL	<MDL		0.197	0.0986	0.0988
Perfluoroether Carboxylic Acids	ADONA	<MDL	<MDL	<MDL		0.197	0.0986	0.0988
	HFPO-DA	<MDL	<MDL	<MDL		0.197	0.0986	0.0988
Chlorinated Polyfluoroalkyl Ether	9Cl-PF3ONS	<MDL	<MDL	<MDL		0.197	0.0986	0.0988
	11Cl-PF3OUdS	*-*1	*-*1	*-*1		0.197	0.0986	0.0988
General Chemistry								
Total Volatile Solids	%	22.3	19.3	17.7		0.500	0.500	0.500
Solids, total	%	100	101	99.2		0.500	0.500	0.500

J: Result is less than the RL but greater than or equal to the MDL and the concentration is an approximate value.

B: Compound was found in the blank and sample.

I: Value is EMPC (estimated maximum possible concentration).

*-: LCS and/or LCSD is outside acceptance limits, low biased.

R: Rejected sample

<MDL: Non Detectable

Note that all analytes were subject to H or H3 qualifiers, indicating the sample was prepped or analyzed or received beyond the specific holding time. The sample does not meet regulatory requirements

Table S-8 Flue gas (Sample Point 4 Front Half) targeted liquid or gas chromatographic–mass spectrometric results compared to quality control results (Matrix: Flue Gas). The Field Blank Train (FBT) uses glassware previously used at the current site from a completed run. The Proof Blank Train (PBT) uses glassware before it has been used for sampling. Complete description of FBT and PBT in the OTM-45 method. Units: ng/sample.

Family	Acronym	Front Half			Field Blank Train (FBT) Front Half	Proof Blank Train (PBT) Front Half	Media check XAD Does Not Apply	Media Check Filter
		R1	R2	R3				
	PFBA	CI	CI	CI	<MDL	<MDL		<MDL
	PFPeA	1.95	1.45	3.10	<MDL	<MDL		<MDL
	PFHxA	3.72	3.42	6.06	<MDL	<MDL		<MDL
	PFHpA	2.43	1.41	3.61	<MDL	<MDL		<MDL
	PFOA	9.12	7.61	10.5	<MDL	<MDL		<MDL
	PFNA	1.98	1.17	1.41	<MDL	<MDL		<MDL
	PFDA	1.28	J	J	<MDL	<MDL		<MDL
	PFUnA	J	J	J	<MDL	<MDL		<MDL
	PFDoA	J	J	J	<MDL	<MDL		<MDL
	PFTrDA	J	J	<MDL	<MDL	<MDL		<MDL
	PFTeA	J	<MDL	<MDL	<MDL	<MDL		<MDL
	PFHxDA	<MDL	J	<MDL	<MDL	<MDL		<MDL
Perfluorocarboxylic Acids (PFCAs)	PFODA	<MDL	<MDL	<MDL	<MDL	<MDL		<MDL
	PFBS	<MDL	I	<MDL	<MDL	<MDL		<MDL
	PFPeS	<MDL	ICI	I	<MDL	<MDL		<MDL
	PFHxS	<MDL	<MDL	<MDL	<MDL	<MDL		<MDL
	PFHpS	<MDL	<MDL	<MDL	<MDL	<MDL		<MDL
	PFOS	<MDL	<MDL	<MDL	<MDL	<MDL		<MDL
	PFNS	<MDL	JI	<MDL	<MDL	<MDL		<MDL
	PFDS	<MDL	ICI	JI	<MDL	<MDL		<MDL
Perfluorosulfonates (PFSAs)	PFDoS	<MDL	<MDL	<MDL	<MDL	<MDL		<MDL
	FOSA	<MDL	<MDL	<MDL	<MDL	<MDL		<MDL
	NEtFOSA	<MDL	<MDL	<MDL	<MDL	<MDL		<MDL
	NMeFOSA	<MDL	<MDL	<MDL	<MDL	<MDL		<MDL
	NMeFOSAA	<MDL	<MDL	<MDL	<MDL	<MDL		<MDL
	NEtFOSAA	J	J	J	<MDL	<MDL		<MDL
	NMeFOSE	13.1	9.60	5.63	<MDL	<MDL		<MDL
Sulfonamides	NEtFOSE	J	J	J	<MDL	<MDL		<MDL
	4:2 FTS	<MDL	<MDL	<MDL	<MDL	<MDL		<MDL
	6:2 FTS	5.05	J	44.2	<MDL	<MDL		<MDL
	8:2 FTS	<MDL	<MDL	<MDL	<MDL	<MDL		<MDL
Fluorotelomers	10:2 FTS	<MDL	<MDL	<MDL	<MDL	<MDL		<MDL
	ADONA	<MDL	<MDL	<MDL	<MDL	<MDL		<MDL
Polyfluoroalkyl Substances	HFPO-DA	<MDL	<MDL	<MDL	<MDL	<MDL		<MDL
	9Cl-PF3ONS	<MDL	<MDL	<MDL	<MDL	<MDL		<MDL
Chlorinated Polyfluoroalkyl Substances	11Cl-PF3OUdS	<MDL	<MDL	<MDL	<MDL	<MDL		<MDL
Fluorotelomer Unsaturated Carboxylic Acids	6:2 FTUCA	<MDL	JI	<MDL	<MDL	<MDL		<MDL
	7:3 FTCA	J	*+	*+	*+	*+		*+
	10:2 FTCA	<MDL	<MDL	<MDL	<MDL	<MDL		<MDL
Fluorotelomer Carboxylic Acids	8:2 FTCA	<MDL	<MDL	<MDL	<MDL	<MDL		<MDL
Ethoxylated Substances	PFEESA	<MDL	<MDL	<MDL	<MDL	<MDL		<MDL
Fluorotelomer Unsaturated Carboxylic Acids	8:2 FTUCA	<MDL	<MDL	<MDL	<MDL	<MDL		<MDL
Methoxylated Substances	PFMPA	<MDL	<MDL	<MDL	<MDL	<MDL		<MDL
Oxahexanoic Substances	PFMBA	<MDL	<MDL	<MDL	<MDL	<MDL		<MDL
	5:3 FTCA	*+	*+	*+	*+	*+		*+
	6:2 FTCA	<MDL	<MDL	<MDL	<MDL	<MDL		<MDL
Fluorotelomer Carboxylic Acids	3:3 FTCA	<MDL	<MDL	<MDL	<MDL	<MDL		<MDL
Ethoxylated Substances	PFECHS	<MDL	<MDL	<MDL	<MDL	<MDL		<MDL
Dioxaheptanoic Substances	NFDHA	<MDL	<MDL	<MDL	<MDL	<MDL		<MDL

J: Result is less than the RL but greater than or equal to the MDL and the concentration is an approximate value.
I: Value is EMPC (estimated maximum possible concentration).
*+: LCS and/or LCSD is outside acceptance limits, high biased.
CI: The peak identified by the data system exhibited chromatographic interference that could not be resolved. There is reason to suspect there may be a high bias.
<MDL: Non Detectable

Table S-9 Flue gas (Sample Point 4 Back Half) targeted liquid or gas chromatographic–mass spectrometric results compared to quality control results (Matrix: Flue Gas). The Field Blank Train (FBT) uses glassware previously used at the current site from a completed run. The Proof Blank Train (PBT) uses glassware before it has been used for sampling. Complete description of FBT and PBT in the OTM-45 method. Units: ng/sample.

Family	Acronym	Back Half			Field Blank Train (FBT) Back Half	Proof Blank Train (PBT) Back Half	Media check XAD	Media Check Filter - Does Not Apply
		R1	R2	R3				
	PFBA	<MDL	20.7	<MDL	CI	CI	<MDL	
	PFPeA	3.28	1.25	2.93	<MDL	<MDL	<MDL	
	PFHxA	7.42	ICI	3.72	J	<MDL	<MDL	
	PFHpA	4.96	3.73	3.67	<MDL	<MDL	<MDL	
	PFOA	9.48	6.54	8.76	4.31	J	<MDL	
	PFNA	1.13	<MDL	1.02	<MDL	<MDL	<MDL	
	PFDA	J	J	J	J	<MDL	<MDL	
	PFUnA	J	<MDL	1.08	<MDL	<MDL	<MDL	
	PFDoA	R	R	J	<MDL	<MDL	<MDL	
	PFTrDA	R	R	<MDL	<MDL	<MDL	<MDL	
	PFTeA	R	R	R	R	R	R	
	PFHxDA	R	R	R	R	R	R	
Perfluorocarboxylic Acids (PFCAs)	PFODA	*-R	*-R	*-R	*-R	*-R	*-R	
	PFBS	J	<MDL	JI	<MDL	<MDL	<MDL	
	PFPeS	JI	ICI	JI	<MDL	<MDL	<MDL	
	PFHxS	<MDL	<MDL	<MDL	<MDL	<MDL	<MDL	
	PFHpS	<MDL	I	<MDL	<MDL	<MDL	<MDL	
	PFOS	<MDL	<MDL	J	<MDL	<MDL	<MDL	
	PFNS	<MDL	<MDL	<MDL	<MDL	<MDL	<MDL	
	PFDS	<MDL	<MDL	<MDL	<MDL	<MDL	<MDL	
Perfluorosulfonates (PFSAs)	PFDoS	*-	*-	*-	*-	*-	*-	
	FOSA	R	R	<MDL	<MDL	<MDL	<MDL	
	NEtFOSA	R	R	R	<MDL	<MDL	<MDL	
	NMeFOSA	R	R	R	<MDL	<MDL	<MDL	
	NMeFOSAA	<MDL	<MDL	<MDL	<MDL	<MDL	<MDL	
	NEtFOSAA	J	J	<MDL	<MDL	<MDL	<MDL	
	NMeFOSE	R	R	<MDL	<MDL	<MDL	<MDL	
Sulfonamides	NEtFOSE	0	R	<MDL	<MDL	<MDL	<MDL	
	4:2 FTS	<MDL	<MDL	<MDL	<MDL	<MDL	<MDL	
	6:2 FTS	<MDL	<MDL	<MDL	<MDL	<MDL	<MDL	
	8:2 FTS	<MDL	<MDL	<MDL	<MDL	<MDL	<MDL	
Fluorotelomers	10:2 FTS	R	R	<MDL	<MDL	<MDL	<MDL	
	ADONA	<MDL	<MDL	<MDL	<MDL	<MDL	<MDL	
Polyfluoroalkyl Substances	HFPO-DA	<MDL	<MDL	<MDL	<MDL	<MDL	<MDL	
	9Cl-PF3ONS	<MDL	<MDL	<MDL	<MDL	<MDL	<MDL	
Chlorinated Polyfluoroalkyl Substances	11Cl-PF3OUdS	<MDL	<MDL	<MDL	<MDL	<MDL	<MDL	
Fluorotelomer Unsaturated Carboxylic Acids	6:2 FTUCA	<MDL	<MDL	<MDL	<MDL	<MDL	<MDL	
	7:3 FTCA	*+	*+	*+	*+	*+	*+	
	10:2 FTCA	JIR	R	<MDL	<MDL	<MDL	<MDL	
Fluorotelomer Carboxylic Acids	8:2 FTCA	<MDL	<MDL	<MDL	<MDL	<MDL	<MDL	
Ethoxylated Substances	PFEESA	<MDL	<MDL	<MDL	<MDL	<MDL	<MDL	
Fluorotelomer Unsaturated Carboxylic Acids	8:2 FTUCA	<MDL	<MDL	<MDL	<MDL	<MDL	<MDL	
Methoxylated Substances	PFMPA	<MDL	<MDL	<MDL	<MDL	<MDL	<MDL	
Oxahexanoic Substances	PFMBA	<MDL	<MDL	<MDL	<MDL	<MDL	<MDL	
	5:3 FTCA	*+	*+	*+	*+	*+	*+	
	6:2 FTCA	<MDL	<MDL	<MDL	<MDL	<MDL	<MDL	
Fluorotelomer Carboxylic Acids	3:3 FTCA	J	<MDL	<MDL	<MDL	<MDL	<MDL	
Ethoxylated Substances	PFECHS	<MDL	<MDL	<MDL	<MDL	<MDL	<MDL	
Dioxaheptanoic Substances	NFDHA	<MDL	<MDL	<MDL	<MDL	<MDL	<MDL	

J: Result is less than the RL but greater than or equal to the MDL and the concentration is an approximate value.
I: Value is EMPC (estimated maximum possible concentration).
*-: LCS and/or LCSD is outside acceptance limits, low biased.
*+: LCS and/or LCSD is outside acceptance limits, high biased.
CI: The peak identified by the data system exhibited chromatographic interference that could not be resolved. There is reason to suspect there may be a high bias.
<MDL: Non Detectable

Table S-10 Flue gas (Sample Point 4 Impinger Condensate) targeted liquid or gas chromatographic–mass spectrometric results compared to quality control results (Matrix: Flue Gas). The Field Blank Train (FBT) uses glassware previously used at the current site from a completed run. The Proof Blank Train (PBT) uses glassware before it has been used for sampling. Complete description of FBT and PBT in the OTM-45 method. Units: ng/sample.

		Impinger Condensate				Field Blank Train (FBT) Impringer Condensate	Proof Blank Train (PBT) Impringer Condensate	Media check XAD-Does Not Apply	Media Check Filter - Does Not Apply
Family	Acronym	R1	R2	R3					
	PFBA	B	BCI	B		<MDL	<MDL		
	PFPeA	1.23	0.634	J		<MDL	<MDL		
	PFHxA	J	J	<MDL		<MDL	<MDL		
	PFHpA	<MDL	<MDL	<MDL		<MDL	<MDL		
	PFOA	J	J	J		<MDL	<MDL		
	PFNA	<MDL	<MDL	<MDL		<MDL	<MDL		
	PFDA	<MDL	<MDL	<MDL		<MDL	<MDL		
	PFUnA	<MDL	<MDL	<MDL		<MDL	<MDL		
	PFDoA	<MDL	<MDL	<MDL		<MDL	<MDL		
	PFTrDA	<MDL	<MDL	<MDL		<MDL	<MDL		
	PFTeA	<MDL	<MDL	<MDL		<MDL	<MDL		
	PFHxDA	<MDL	<MDL	<MDL		<MDL	<MDL		
Perfluorocarboxylic Acids (PFCAs)	PFODA	<MDL	<MDL	<MDL		<MDL	<MDL		
	PFBS	JI	JI	JI		<MDL	<MDL		
	PFPeS	<MDL	<MDL	<MDL		<MDL	<MDL		
	PFHxS	<MDL	<MDL	<MDL		<MDL	<MDL		
	PFHpS	<MDL	<MDL	<MDL		<MDL	<MDL		
	PFOS	J	<MDL	J		<MDL	<MDL		
	PFNS	<MDL	<MDL	<MDL		<MDL	<MDL		
	PFDS	<MDL	<MDL	<MDL		<MDL	<MDL		
Perfluorosulfonates (PFSAs)	PFDoS	<MDL	<MDL	<MDL		<MDL	<MDL		
	FOSA	<MDL	<MDL	<MDL		<MDL	<MDL		
	NEtFOSA	<MDL	<MDL	<MDL		<MDL	<MDL		
	NMeFOSA	<MDL	<MDL	<MDL		<MDL	<MDL		
	NMeFOSAA	<MDL	<MDL	<MDL		<MDL	<MDL		
	NEtFOSAA	<MDL	<MDL	<MDL		<MDL	<MDL		
	NMeFOSE	<MDL	<MDL	<MDL		<MDL	<MDL		
Sulfonamides	NEtFOSE	<MDL	<MDL	<MDL		<MDL	<MDL		
	4:2 FTS	<MDL	<MDL	<MDL		<MDL	<MDL		
	6:2 FTS	<MDL	<MDL	<MDL		<MDL	<MDL		
	8:2 FTS	<MDL	<MDL	<MDL		<MDL	<MDL		
Fluorotelomers	10:2 FTS	<MDL	<MDL	<MDL		<MDL	<MDL		
	ADONA	<MDL	<MDL	<MDL		<MDL	<MDL		
Polyfluoroalkyl Substances	HFPO-DA	<MDL	<MDL	<MDL		<MDL	<MDL		
	9Cl-PF3ONS	<MDL	<MDL	<MDL		<MDL	<MDL		
Chlorinated Polyfluoroalkyl Substances	11Cl-PF3OUdS	<MDL	<MDL	<MDL		<MDL	<MDL		
Fluorotelomer Unsaturated Carboxylic Acids	6:2 FTUCA	<MDL	<MDL	<MDL		<MDL	<MDL		
	7:3 FTCA	<MDL	<MDL	<MDL		<MDL	<MDL		
	10:2 FTCA	<MDL	<MDL	<MDL		<MDL	<MDL		
Fluorotelomer Carboxylic Acids	8:2 FTCA	<MDL	<MDL	<MDL		<MDL	<MDL		
Ethoxylated Substances	PFEESA	<MDL	<MDL	<MDL		<MDL	<MDL		
Fluorotelomer Unsaturated Carboxylic Acids	8:2 FTUCA	<MDL	<MDL	<MDL		<MDL	<MDL		
Methoxylated Substances	PFMPA	<MDL	<MDL	<MDL		<MDL	<MDL		
Oxahexanoic Substances	PFMBA	<MDL	<MDL	<MDL		<MDL	<MDL		
	5:3 FTCA	<MDL	<MDL	<MDL		<MDL	<MDL		
	6:2 FTCA	<MDL	<MDL	<MDL		<MDL	<MDL		
Fluorotelomer Carboxylic Acids	3:3 FTCA	<MDL	<MDL	<MDL		<MDL	<MDL		
Ethoxylated Substances	PFECHS	<MDL	<MDL	<MDL		<MDL	<MDL		
Dioxaheptanoic Substances	NFDHA	<MDL	<MDL	<MDL		<MDL	<MDL		

J: Result is less than the RL but greater than or equal to the MDL and the concentration is an approximate value.
B: Compound was found in the blank and sample.
I: Value is EMPC (estimated maximum possible concentration).
CI: The peak identified by the data system exhibited chromatographic interference that could not be resolved. There is reason to suspect there may be a high bias.
<MDL: Non Detectable

Table S-11 Flue gas (Sample Point 4 Breakthrough XAD resin) targeted liquid or gas chromatographic–mass spectrometric results compared to quality control results (Matrix: Flue Gas). The Field Blank Train (FBT) uses glassware previously used at the current site from a completed run. The Proof Blank Train (PBT) uses glassware before it has been used for sampling. Complete description of FBT and PBT in the OTM-45 method. Units: ng/sample.

		Breakthrough XAD resin			Field Blank Train (FBT) Breakthrough XAD resin	Proof Blank Train (PBT) Breakthrough XAD resin	Media check XAD	Media Check Filter - Does Not Apply
Family	Acronym	R1	R2	R3				
	PFBA	CI	CI	<MDL	CI	CI	<MDL	
	PFPeA	<MDL	<MDL	<MDL	<MDL	<MDL	<MDL	
	PFHxA	<MDL	<MDL	J	<MDL	<MDL	<MDL	
	PFHpA	<MDL	<MDL	<MDL	<MDL	<MDL	<MDL	
	PFOA	<MDL	1.28	1.18	J	J	<MDL	
	PFNA	<MDL	<MDL	<MDL	<MDL	<MDL	<MDL	
	PFDA	<MDL	<MDL	<MDL	<MDL	<MDL	<MDL	
	PFUnA	<MDL	<MDL	<MDL	<MDL	<MDL	<MDL	
	PFDoA	R	R	<MDL	<MDL	<MDL	<MDL	
	PFTrDA	R	R	<MDL	<MDL	<MDL	<MDL	
	PFTeA	R	R	R	R	R	R	
	PFHxDA	R	R	R	R	R	R	
Perfluorocarboxylic Acids (PFCAs)	PFODA	*-R	*-R	*-R	*-R	*-R	*-R	
	PFBS	<MDL	<MDL	<MDL	<MDL	<MDL	<MDL	
	PFPeS	<MDL	<MDL	<MDL	<MDL	<MDL	<MDL	
	PFHxS	<MDL	<MDL	<MDL	<MDL	<MDL	<MDL	
	PFHpS	<MDL	<MDL	<MDL	<MDL	<MDL	<MDL	
	PFOS	<MDL	JI	<MDL	<MDL	<MDL	<MDL	
	PFNS	<MDL	<MDL	<MDL	<MDL	<MDL	<MDL	
	PFDS	<MDL	<MDL	<MDL	<MDL	<MDL	<MDL	
Perfluorosulfonates (PFSAs)	PFDoS	*-	*-	*-	*-	*-	*-	
	FOSA	<MDL	<MDL	<MDL	<MDL	<MDL	<MDL	
	NEtFOSA	<MDL	<MDL	R	<MDL	<MDL	<MDL	
	NMeFOSA	<MDL	<MDL	<MDL	<MDL	<MDL	<MDL	
	NMeFOSAA	<MDL	<MDL	<MDL	<MDL	<MDL	<MDL	
	NEtFOSAA	<MDL	<MDL	J	<MDL	<MDL	<MDL	
	NMeFOSE	<MDL	<MDL	<MDL	<MDL	<MDL	<MDL	
Sulfonamides	NEtFOSE	R	<MDL	<MDL	<MDL	<MDL	<MDL	
	4:2 FTS	<MDL	<MDL	<MDL	<MDL	<MDL	<MDL	
	6:2 FTS	<MDL	<MDL	<MDL	<MDL	<MDL	<MDL	
	8:2 FTS	<MDL	<MDL	<MDL	<MDL	<MDL	<MDL	
Fluorotelomers	10:2 FTS	R	R	<MDL	<MDL	<MDL	<MDL	
	ADONA	<MDL	<MDL	<MDL	<MDL	<MDL	<MDL	
Polyfluoroalkyl Substances	HFPO-DA	<MDL	<MDL	<MDL	<MDL	<MDL	<MDL	
	9Cl-PF3ONS	<MDL	<MDL	<MDL	<MDL	<MDL	<MDL	
Chlorinated Polyfluoroalkyl Substances	11Cl-PF3OUdS	<MDL	<MDL	<MDL	<MDL	<MDL	<MDL	
Fluorotelomer Unsaturated Carboxylic Acids	6:2 FTUCA	<MDL	<MDL	<MDL	<MDL	<MDL	<MDL	
	7:3 FTCA	*+	*+	*+	*+	*+	*+	
	10:2 FTCA	<MDL	<MDL	<MDL	<MDL	<MDL	<MDL	
Fluorotelomer Carboxylic Acids	8:2 FTCA	<MDL	<MDL	<MDL	<MDL	<MDL	<MDL	
Ethoxylated Substances	PFEESA	<MDL	<MDL	<MDL	<MDL	<MDL	<MDL	
Fluorotelomer Unsaturated Carboxylic Acids	8:2 FTUCA	<MDL	<MDL	<MDL	<MDL	<MDL	<MDL	
Methoxylated Substances	PFMPA	<MDL	<MDL	<MDL	<MDL	<MDL	<MDL	
Oxahexanoic Substances	PFMBA	<MDL	<MDL	<MDL	<MDL	<MDL	<MDL	
	5:3 FTCA	*+	*+	*+	*+	*+	*+	
	6:2 FTCA	<MDL	<MDL	<MDL	<MDL	<MDL	<MDL	
Fluorotelomer Carboxylic Acids	3:3 FTCA	<MDL	<MDL	<MDL	<MDL	<MDL	<MDL	
Ethoxylated Substances	PFECHS	<MDL	<MDL	<MDL	<MDL	<MDL	<MDL	
Dioxaheptanoic Substances	NFDHA	<MDL	<MDL	<MDL	<MDL	<MDL	<MDL	

J: Result is less than the RL but greater than or equal to the MDL and the concentration is an approximate value.
I: Value is EMPC (estimated maximum possible concentration).
*+: LCS and/or LCSD is outside acceptance limits, high biased.
<MDL: Non Detectable

Table S-12 Flue gas (Sample Point 4) targeted liquid or gas chromatographic–mass spectrometric reporting limits (RL) (Matrix: Flue Gas). Units: ng/sample.

		Front Half			Back Half			Impinger			Breakthrough XAD		
Family	Acronym	R1	R2	R3	R1	R2	R3	R1	R2	R3	R1	R2	R3
	PFBA	1.95	2.00	2.00	10.0	10.0	10.0	0.500	0.500	0.500	10.0	10.0	10.0
	PFPeA	0.977	1.00	1.00	1.00	1.00	1.00	0.500	0.500	0.500	1.00	1.00	1.00
	PFHxA	0.977	1.00	1.00	1.00	1.00	1.00	0.500	0.500	0.500	1.00	1.00	1.00
	PFHpA	0.977	1.00	1.00	3.00	3.00	3.00	0.500	0.500	0.500	3.00	3.00	3.00
	PFOA	0.977	1.00	1.00	1.00	1.00	1.00	0.500	0.500	0.500	1.00	1.00	1.00
	PFNA	0.977	1.00	1.00	1.00	1.00	1.00	0.500	0.500	0.500	1.00	1.00	1.00
	PFDA	0.977	1.00	1.00	1.00	1.00	1.00	0.500	0.500	0.500	1.00	1.00	1.00
	PFUnA	0.977	1.00	1.00	1.00	1.00	1.00	0.500	0.500	0.500	1.00	1.00	1.00
	PFDoA	0.977	1.00	1.00	1.00	1.00	1.00	0.500	0.500	0.500	1.00	1.00	1.00
	PFTrDA	0.977	1.00	1.00	1.00	1.00	1.00	0.500	0.500	0.500	1.00	1.00	1.00
	PFTeA	0.977	1.00	1.00	1.00	1.00	1.00	0.500	0.500	0.500	1.00	1.00	1.00
	PFHxDA	0.977	1.00	1.00	1.00	1.00	1.00	0.500	0.500	0.500	1.00	1.00	1.00
Perfluorocarboxylic Acids (PFCAs)	PFODA	0.977	1.00	1.00	1.00	1.00	1.00	0.500	0.500	0.500	1.00	1.00	1.00
	PFBS	0.977	1.00	1.00	1.00	1.00	1.00	0.500	0.500	0.500	1.00	1.00	1.00
	PFPeS	0.977	1.00	1.00	1.00	1.00	1.00	0.500	0.500	0.500	1.00	1.00	1.00
	PFHxS	0.977	1.00	1.00	1.00	1.00	1.00	0.500	0.500	0.500	1.00	1.00	1.00
	PFHpS	0.977	1.00	1.00	1.00	1.00	1.00	0.500	0.500	0.500	1.00	1.00	1.00
	PFOS	0.977	1.00	1.00	1.00	1.00	1.00	0.500	0.500	0.500	1.00	1.00	1.00
	PFNS	0.977	1.00	1.00	1.00	1.00	1.00	0.500	0.500	0.500	1.00	1.00	1.00
	PFDS	0.977	1.00	1.00	1.00	1.00	1.00	0.500	0.500	0.500	1.00	1.00	1.00
Perfluorosulfonates (PFSAs)	PFDoS	0.977	1.00	1.00	1.00	1.00	1.00	0.500	0.500	0.500	1.00	1.00	1.00
	FOSA	0.977	1.00	1.00	1.00	1.00	1.00	0.500	0.500	0.500	1.00	1.00	1.00
	NEtFOSA	0.977	1.00	1.00	1.00	1.00	1.00	0.500	0.500	0.500	1.00	1.00	1.00
	NMeFOSA	0.977	1.00	1.00	1.00	1.00	1.00	0.500	0.500	0.500	1.00	1.00	1.00
	NMeFOSAA	0.977	1.00	1.00	1.00	1.00	1.00	0.500	0.500	0.500	1.00	1.00	1.00
	NEtFOSAA	0.977	1.00	1.00	1.00	1.00	1.00	0.500	0.500	0.500	1.00	1.00	1.00
	NMeFOSE	4.89	5.00	5.00	20.0	20.0	20.0	0.500	0.500	0.500	20.0	20.0	20.0
Sulfonamides	NEtFOSE	0.977	1.00	1.00	0	1.00	1.00	0.500	0.500	0.500	1.00	1.00	1.00
	4:2 FTS	0.977	1.00	1.00	1.00	1.00	1.00	0.500	0.500	0.500	1.00	1.00	1.00
	6:2 FTS	4.89	5.00	5.00	10.0	10.0	10.0	0.500	0.500	0.500	10.0	10.0	10.0
	8:2 FTS	0.977	1.00	1.00	1.00	1.00	1.00	0.500	0.500	0.500	1.00	1.00	1.00
Fluorotelomers	10:2 FTS	0.977	1.00	1.00	1.00	1.00	1.00	0.500	0.500	0.500	1.00	1.00	1.00
	ADONA	0.977	1.00	1.00	2.00	2.00	2.00	0.500	0.500	0.500	2.00	2.00	2.00
Polyfluoroalkyl Substances	HFPO-DA	4.89	5.00	5.00	20.0	20.0	20.0	0.500	0.500	0.500	20.0	20.0	20.0
	9Cl-PF3ONS	0.977	1.00	1.00	1.00	1.00	1.00	0.500	0.500	0.500	1.00	1.00	1.00
Chlorinated Polyfluoroalkyl Substances	11Cl-PF3OUdS	0.977	1.00	1.00	1.00	1.00	1.00	0.500	0.500	0.500	1.00	1.00	1.00
Fluorotelomer Unsaturated Carboxylic Acids	6:2 FTUCA	0.977	1.00	1.00	1.00	1.00	1.00	0.500	0.500	0.500	1.00	1.00	1.00
	7:3 FTCA	0.977	1.00	1.00	1.00	1.00	1.00	0.500	0.500	0.500	1.00	1.00	1.00
	10:2 FTCA	0.977	1.00	1.00	1.00	1.00	1.00	0.500	0.500	0.500	1.00	1.00	1.00
Fluorotelomer Carboxylic Acids	8:2 FTCA	0.977	1.00	1.00	1.00	1.00	1.00	0.500	0.500	0.500	1.00	1.00	1.00
Ethoxylated Substances	PFEESA	0.977	1.00	1.00	1.00	1.00	1.00	0.500	0.500	0.500	1.00	1.00	1.00
Fluorotelomer Unsaturated Carboxylic Acids	8:2 FTUCA	0.977	1.00	1.00	1.00	1.00	1.00	0.500	0.500	0.500	1.00	1.00	1.00
Methoxylated Substances	PFMPA	0.977	1.00	1.00	1.00	1.00	1.00	0.500	0.500	0.500	1.00	1.00	1.00
Oxahexanoic Substances	PFMBA	0.977	1.00	1.00	1.00	1.00	1.00	0.500	0.500	0.500	1.00	1.00	1.00
	5:3 FTCA	0.977	1.00	1.00	1.00	1.00	1.00	0.500	0.500	0.500	1.00	1.00	1.00
	6:2 FTCA	0.977	1.00	1.00	1.00	1.00	1.00	0.500	0.500	0.500	1.00	1.00	1.00
Fluorotelomer Carboxylic Acids	3:3 FTCA	0.977	1.00	1.00	1.00	1.00	1.00	0.500	0.500	0.500	1.00	1.00	1.00
Ethoxylated Substances	PFECHS	0.977	1.00	1.00	1.00	1.00	1.00	0.500	0.500	0.500	1.00	1.00	1.00
Dioxaheptanoic Substances	NFDHA	0.977	1.00	1.00	1.00	1.00	1.00	0.500	0.500	0.500	1.00	1.00	1.00

Table S-13 Flue gas (Sample Point 4) targeted liquid or gas chromatographic–mass spectrometric quality control (QC) reporting limits (RL) (Matrix: Flue Gas). Units: ng/sample.

		Field Bland Train (FBT) RL				Proof Blank Train (PBT) RL				Media Check XAD RL	Media Check filter RL
Family	Acronym	Front Half	Back Half	Impinger Condensate	Breakthrough XAD Resin	Front Half	Back Half	Impinger Condensate	Breakthrough XAD Resin		
	PFBA	2.00	10.0	0.500	10.0	1.97	10.0	0.500	10.0	10.0	2.00
	PFPeA	1.00	1.00	0.500	1.00	0.985	1.00	0.500	1.00	1.00	1.00
	PFHxA	1.00	1.00	0.500	1.00	0.985	1.00	0.500	1.00	1.00	1.00
	PFHpA	1.00	3.00	0.500	3.00	0.985	3.00	0.500	3.00	3.00	1.00
	PFOA	1.00	1.00	0.500	1.00	0.985	1.00	0.500	1.00	1.00	1.00
	PFNA	1.00	1.00	0.500	1.00	0.985	1.00	0.500	1.00	1.00	1.00
	PFDA	1.00	1.00	0.500	1.00	0.985	1.00	0.500	1.00	1.00	1.00
	PFUnA	1.00	1.00	0.500	1.00	0.985	1.00	0.500	1.00	1.00	1.00
	PFDoA	1.00	1.00	0.500	1.00	0.985	1.00	0.500	1.00	1.00	1.00
	PFTrDA	1.00	1.00	0.500	1.00	0.985	1.00	0.500	1.00	1.00	1.00
	PFTeA	1.00	1.00	0.500	1.00	0.985	1.00	0.500	1.00	1.00	1.00
	PFHxDA	1.00	1.00	0.500	1.00	0.985	1.00	0.500	1.00	1.00	1.00
Perfluorocarboxylic Acids (PFCAs)	PFODA	1.00	1.00	0.500	1.00	0.985	1.00	0.500	1.00	1.00	1.00
	PFBS	1.00	1.00	0.500	1.00	0.985	1.00	0.500	1.00	1.00	1.00
	PFPeS	1.00	1.00	0.500	1.00	0.985	1.00	0.500	1.00	1.00	1.00
	PFHxS	1.00	1.00	0.500	1.00	0.985	1.00	0.500	1.00	1.00	1.00
	PFHpS	1.00	1.00	0.500	1.00	0.985	1.00	0.500	1.00	1.00	1.00
	PFOS	1.00	1.00	0.500	1.00	0.985	1.00	0.500	1.00	1.00	1.00
	PFNS	1.00	1.00	0.500	1.00	0.985	1.00	0.500	1.00	1.00	1.00
	PFDS	1.00	1.00	0.500	1.00	0.985	1.00	0.500	1.00	1.00	1.00
Perfluorosulfonates (PFSAs)	PFDoS	1.00	1.00	0.500	1.00	0.985	1.00	0.500	1.00	1.00	1.00
	FOSA	1.00	1.00	0.500	1.00	0.985	1.00	0.500	1.00	1.00	1.00
	NEtFOSA	1.00	1.00	0.500	1.00	0.985	1.00	0.500	1.00	1.00	1.00
	NMeFOSA	1.00	1.00	0.500	1.00	0.985	1.00	0.500	1.00	1.00	1.00
	NMeFOSAA	1.00	1.00	0.500	1.00	0.985	1.00	0.500	1.00	1.00	1.00
	NEtFOSAA	1.00	1.00	0.500	1.00	0.985	1.00	0.500	1.00	1.00	1.00
	NMeFOSE	5.00	20.0	0.500	20.0	4.93	20.0	0.500	20.0	20.0	5.00
Sulfonamides	NEtFOSE	1.00	1.00	0.500	1.00	0.985	1.00	0.500	1.00	1.00	1.00
	4:2 FTS	1.00	1.00	0.500	1.00	0.985	1.00	0.500	1.00	1.00	1.00
	6:2 FTS	5.00	10.0	0.500	10.0	4.93	10.0	0.500	10.0	10.0	5.00
	8:2 FTS	1.00	1.00	0.500	1.00	0.985	1.00	0.500	1.00	1.00	1.00
Fluorotelomers	10:2 FTS	1.00	1.00	0.500	1.00	0.985	1.00	0.500	1.00	1.00	1.00
	ADONA	1.00	2.00	0.500	2.00	0.985	2.00	0.500	2.00	2.00	1.00
Polyfluoroalkyl Substances	HFPO-DA	5.00	20.0	0.500	20.0	4.93	20.0	0.500	20.0	20.0	5.00
	9Cl-PF3ONS	1.00	1.00	0.500	1.00	0.985	1.00	0.500	1.00	1.00	1.00
Chlorinated Polyfluoroalkyl Substances	11Cl-PF3OUdS	1.00	1.00	0.500	1.00	0.985	1.00	0.500	1.00	1.00	1.00
Fluorotelomer Unsaturated Carboxylic Acids	6:2 FTUCA	1.00	1.00	0.500	1.00	0.985	1.00	0.500	1.00	1.00	1.00
	7:3 FTCA	1.00	1.00	0.500	1.00	0.985	1.00	0.500	1.00	1.00	1.00
	10:2 FTCA	1.00	1.00	0.500	1.00	0.985	1.00	0.500	1.00	1.00	1.00
Fluorotelomer Carboxylic Acids	8:2 FTCA	1.00	1.00	0.500	1.00	0.985	1.00	0.500	1.00	1.00	1.00
Ethoxylated Substances	PFEESA	1.00	1.00	0.500	1.00	0.985	1.00	0.500	1.00	1.00	1.00
Fluorotelomer Unsaturated Carboxylic Acids	8:2 FTUCA	1.00	1.00	0.500	1.00	0.985	1.00	0.500	1.00	1.00	1.00
Methoxylated Substances	PFMPA	1.00	1.00	0.500	1.00	0.985	1.00	0.500	1.00	1.00	1.00
Oxahexanoic Substances	PFMBA	1.00	1.00	0.500	1.00	0.985	1.00	0.500	1.00	1.00	1.00
	5:3 FTCA	1.00	1.00	0.500	1.00	0.985	1.00	0.500	1.00	1.00	1.00
	6:2 FTCA	1.00	1.00	0.500	1.00	0.985	1.00	0.500	1.00	1.00	1.00
Fluorotelomer Carboxylic Acids	3:3 FTCA	1.00	1.00	0.500	1.00	0.985	1.00	0.500	1.00	1.00	1.00
Ethoxylated Substances	PFECHS	1.00	1.00	0.500	1.00	0.985	1.00	0.500	1.00	1.00	1.00
Dioxaheptanoic Substances	NFDHA	1.00	1.00	0.500	1.00	0.985	1.00	0.500	1.00	1.00	1.00

References

Buck, R. C., Franklin, J., Berger, U., Conder, J. M., Cousins, I. T., Voogt, P. De, Jensen, A. A., Kannan, K., Mabury, S. A., & van Leeuwen, S. P. J. (2011). Perfluoroalkyl and polyfluoroalkyl substances in the environment: Terminology, classification, and origins. Integrated Environmental Assessment and Management, 7(4), 513–541. https://doi.org/10.1002/ieam.258

ITRC (Interstate Technology & Regulatory Council). (2022a). Environmental Data Management Best 1620 Practices (EDMBP) Team Materials. Washington, D.C.: Interstate Technology & Regulatory Council, 1621 EDMBP Team. https://edm-1.itrcweb.org/ 1622

ITRC (Interstate Technology & Regulatory Council). (2022b). PFAS Technical and Regulatory Guidance 1623 Document and Fact Sheets PFAS-1. Section 11.3. Washington, D.C.: Interstate Technology & 1624 Regulatory Council, PFAS Team. https://pfas-1.itrcweb.org/

USEPA. (1995). Development of compliance levels from analytical detection and quantitation levels, EPA 1658 Document Number: PB95-216321. Retrieved from 1659 https://nepis.epa.gov/Exe/ZyPDF.cgi?Dockey=9101IMKK.PDF

USEPA. (2017a). Method 2 — Determination of Stack Gas Velocity and Volumetric Flow Rate (Type S 1661 Pitot Tube). Retrieved from https://www.epa.gov/sites/default/files/2017- 1662 08/documents/method_2.pdf 1663

USEPA. (2017b). Method 3A — Determination of Oxygen and Carbon Dioxide Concentrations in 1664 Emissions from Stationary Sources (Instrumental Analyzer Procedure). Retrieved from 1665 https://www.epa.gov/sites/default/files/2017-08/documents/method_3a.pdf 1666

USEPA. (2017c). Method 4 — Determination of Moisture Content in Stack Gases. Retrieved from 1667 https://www.epa.gov/sites/default/files/2017-08/documents/method_4_0.pdf 1668

USEPA. (2017d). Appendix B—Definition and Procedure for the Determination of the Method Detection 1669 Limit—Revision 2. 40 C.F.R. § 136. Retrieved from https://www.ecfr.gov/current/title-40/chapter1670 I/subchapter-D/part-136#Appendix-B-to-Part-136

USEPA. (2021). Other Test Method 45 (OTM-45) Measurement of Selected Per- and Polyfluorinated Alkyl Substances from Stationary Sources.

USEPA. (2022a). Draft Method 1621—Screening Method for the Determination of Adsorbable Organic 1682 Fluorine (AOF) – Aqueous Matrices by Combustion Ion Chromatography (CIC).

USEPA. (2022b). 3rd Draft Method 1633—Analysis of Per- and Polyfluoroalkyl Substances (PFAS) in 1686 Aqueous, Solid, Biosolids, and Tissue Samples by LC-MS/M. Retrieved from 1687 https://www.epa.gov/system/files/documents/2022- 1688 12/3rd%20Draft%20Method%201633%20December%202022%2012-20-22_508.pdf

USEPA. (2024a). Method 1633 Analysis of Per- and Polyfluoroalkyl Substances (PFAS) in Aqueous, Solid, Biosolids, and Tissue Samples by LC-MS/MS. https://www.epa.gov/system/files/documents/2024-01/method-1633-final-forweb-posting.pdf
